# Medical and Surgical Episodes Among Hospital Participants in the Bundled Payments for Care Improvement–Advanced Program

**DOI:** 10.1001/jamanetworkopen.2024.51792

**Published:** 2024-12-23

**Authors:** Keenan J. Robbins, Jie Zheng, R. J. Waken, E. John Orav, Arnold M. Epstein, Karen E. Joynt Maddox

**Affiliations:** 1 Department of Surgery, Washington University School of Medicine, St Louis, Missouri; 2 Department of Health Policy and Management, Harvard T.H. Chan School of Public Health, Boston, Massachusetts; 3 Biostatistics Division, Institute for Informatics, Data Science, and Biostatistics, Washington University School of Medicine, St Louis, Missouri; 4 Center for Advancing Health Services, Policy & Economics Research, Institute for Public Health, Washington University, St Louis, Missouri; 5 Division of General Internal Medicine and Primary Care, Department of Medicine, Brigham and Women’s Hospital, Boston, Massachusetts; 6 Department of Biostatistics, Harvard T.H. Chan School of Public Health, Boston, Massachusetts; 7 Cardiovascular Division, Department of Medicine, Washington University School of Medicine, St Louis, Missouri

## Abstract

**Importance:**

Hospital participation in the Bundled Payments for Care Improvement–Advanced (BPCI-A) initiative has been associated with modest savings and stable clinical outcomes overall, but it is unknown whether the program performs differently for medical and surgical or procedural (henceforth, surgical) episodes.

**Objective:**

To assess the association of BPCI-A participation with Medicare spending and clinical outcomes for medical and surgical episodes.

**Design, Setting, and Participants:**

This retrospective difference-in-differences cohort study utilized 100% Medicare fee-for-service inpatient claims for episodes initiated between January 1, 2017, and September 30, 2019, and included 90 days of follow-up. The setting was hospitals participating in BPCI-A and matched nonparticipant hospitals. Participants included Medicare beneficiaries admitted for medical or surgical episodes.

**Exposure:**

BPCI-A participation, beginning in 2018.

**Main Outcomes and Measures:**

Changes in 90-day Medicare payments and clinical outcomes (readmissions, mortality, healthy days at home).

**Results:**

The final sample included 2 895 878 episodes; 1 618 172 (55.9%) were female, 324 186 (11.2%) people under age 65 years, 1 354 246 (46.8%) between 65 and 80 years of age, and 1 217 446 (42.0%) over 80 years of age. Patient characteristics at BPCI-A and comparison hospitals were similar. BPCI-A participation was associated with a decrease in payments for medical episodes (−$882 per episode; 95% CI −$1004 to −$760) and surgical episodes (−$587; 95% CI −$850 to −$324) compared with nonparticipant hospitals (absolute difference between change in medical and surgical episodes, −$295; 95% CI −$584 to −$5). BPCI-A participation was associated with a greater increase in healthy days at home and a greater decrease in both skilled nursing facility (SNF) admissions and SNF length of stay overall, but there were no significant differences between medical and surgical episodes in terms of the association of BPCI-A with changes in these outcomes.

**Conclusions and Relevance:**

In this cohort study analyzing the outcomes of the BPCI-A program for medical and surgical episodes, BPCI-A participation was associated with modestly decreased payments for both medical and surgical episodes compared with nonparticipants. Improvements in clinical outcomes associated with BPCI-A participation were also modest for both types of episodes; this study does not support a hypothesis that either medical or surgical episodes are better-suited for episode-based payments.

## Key Points

**Question:**

Did medical and surgical or procedural episodes perform differently in the first year of the Bundled Payments for Care Improvement–Advanced (BPCI-A) program?

**Findings:**

In this cohort study that included 2 895 878 episodes, BPCI-A participation was associated with a decrease in Medicare payments of $882 per episode for medical episodes and $587 per episode for surgical or procedural episodes, a difference of $295 per episode that did not reach statistical significance when compared in absolute terms.

**Meaning:**

The BPCI-A program was associated with modest savings for both medical and surgical episodes.

## Teaser

This cohort study examines whether Bundled Payments for Care Improvement[en dash]Advanced (BPCI-A) participation is associated with Medicare spending and clinical outcomes for medical and surgical episodes among hospitals.

## Introduction

In efforts to reduce health care spending while improving coordination and quality of care, the Centers for Medicare & Medicaid Services (CMS) have introduced several alternative payment models in recent years. One is the Bundled Payments for Care Improvement–Advanced (BPCI-A) model. Launched in October 2018, BPCI-A holds participating institutions accountable based on risk-adjusted target prices for 90-day clinical episodes initiated by a hospital admission or an outpatient procedure. Participants are eligible for financial rewards if Medicare payments per episode are below target prices and certain quality metrics are achieved, but they may incur penalties if episode payments exceed target prices.^1^ The first model year of this voluntary program offered 32 clinical episodes, a mixture of medically and surgically or procedurally managed diagnoses, from which hospitals or group practices could choose to participate.^2^

CMS recently proposed the launch of a new bundled payment model in 2026, the Transforming Episode Accountability Model (TEAM), which includes only surgical episodes: lower extremity joint replacement, surgical treatment of hip and/or femur fracture, spinal fusion, coronary artery bypass graft, and major bowel procedure.^3^ Thus, it is crucial to understand whether there are individual clinical episodes, or broad types of episodes, for which this structure is particularly well-suited. However, this is largely unexamined empirically. In BPCI-A, independent analyses of the first year of the program showed that participation was associated with decreased payments,^4^ but these findings reflect aggregate spending inclusive of all episodes. For BPCI-A’s predecessor—the Bundled Payments for Care Improvement (BPCI) initiative—the effects on payments were variable across episode types. For example, 78% of hospitals saw payments decrease for major joint replacement compared with only 46% for congestive heart failure.^5^ From a clinical perspective, one might assume that discrete instances of care such as a joint replacement^6^ or cancer resection for which operative candidates can be actively selected (effectively homogenizing the patient population and the therapeutic pathway) would be ideally suited to bundled payments. On the other hand, chronic disease states such as heart failure or hypertension, where patients present at more heterogeneous stages of disease and an admission is just one of many events in a more protracted, less predictable course could make episodes less meaningful. Alternatively, the higher complexity of medical patients may introduce more opportunities to streamline care and decrease expenditures while improving clinical outcomes.

In a formal evaluation of the first 2 model years (October 1, 2018, to December 31, 2019) of the BPCI-A program, commissioned by CMS and performed by the Lewin Group, it was reported that surgical episodes were associated with greater decreases in payments than were medical episodes, but only a subset of the 32 clinical episode types in the program were examined (10 medical and 3 surgical).^7^ It remains to be seen whether episode types that share a similar pathophysiology (eg, orthopedic injuries) or a similar care process (eg, surgical intervention in general) are more likely to perform well in a bundled payment model. Such information is potentially high-value for CMS and other payers seeking to deploy these models across the Medicare population. Therefore, in this study, we aimed to determine (1) whether there were changes in payments or clinical outcomes for either medical or surgical episodes in BPCI-A participant vs matched comparison hospitals; (2) if so, whether medical episodes differed from surgical episodes in payments or outcomes; and (3) whether patterns for individual episode types were consistent with the overall findings.

## Methods

### Data

This study was approved by the Human Research Protection Office at the Washington University School of Medicine in St Louis. The requirement for informed consent was waived because the data were deidentified. The Strengthening the Reporting of Observational Studies in Epidemiology (STROBE) reporting guideline was followed.

Participating hospitals were identified using publicly available data. The initial cohort of BPCI-A fee-for-service Medicare participants began in October 2018, hospitals were allowed to exit in early 2019, and a second cohort commenced in January 2020 (after our study period). Hospitals that exited in 2019, as well as those that joined in the latter group, were excluded from the comparison group. All other nonparticipating acute care hospitals paid by Medicare’s inpatient prospective payment system were considered for inclusion. Medicare Severity-Diagnosis Related Groups (MS-DRGs) were used to categorize patients and attribute them to 1 of the 29 available inpatient clinical episodes that made up the program. These episodes were organized into 2 groups: (1) diagnoses that were managed medically, and (2) those that were managed surgically and/or procedurally (eTable 1 in Supplement 1). Index admissions were identified using fee-for-service inpatient claims with a primary DRG on the list of BPCI-A clinical episodes.

A difference-in-differences design was used to compare changes in outcomes of patient episodes between BPCI-A hospitals and matched control hospitals. Patient episodes were initiated January 1, 2017, through September 30, 2019. Claims through December 31, 2019, were used to accrue 90 days of follow-up. The baseline period was defined as January 2017 to September 2018, and the intervention period as October 2018 to September 2019. Patient demographic information was obtained from Medicare enrollment data, and clinical comorbidities were defined using the Medicare Chronic Conditions Data Warehouse. For race and ethnicity, we used the Research Triangle Institute variable, which augments data captured based on self-report at the time of enrollment in social security with information based on surname and residence, and improves sensitivity for Hispanic and Asian individuals.^8^ In Medicare enrollment data, race and ethnicity are captured in a single mutually exclusive variable: American Indian or Alaska Native, Asian or Pacific Islander, Black, Hispanic, White, and other (which is not further defined). Race and ethnicity were included to assess for the generalizability of the findings but were not included in risk adjustment. To ensure accurate collection of comorbidities, patients without continuous enrollment in Medicare Parts A and B during their episode or the year prior were excluded. Beneficiaries who were Medicare-eligible due to end-stage kidney disease were also excluded, per program specifications.

Hospital characteristics were obtained from the American Hospital Association (AHA) 2017 annual survey and were supplemented by data from the Area Health Resources (AHR) File. Of 709 initial BPCI-A participant hospitals, 1 did not match to AHA or AHR data and was excluded. Hospital-episode pairs were matched with a pool of up to 3885 nonparticipant hospital-episode pairs (depending on condition) using 1-to-3 matching based on propensity scores for BPCI-A participation. The variables used for matching were county-level population, for-profit status, median household income, Medicare Advantage penetration, region, teaching status, size of the hospital, hospital system status, urban location, Disproportionate Share Hospital payment, percentage of Medicare patients and condition-specific volumes. Three participant hospitals did not have any matches within a caliper cutoff of 0.2 and were excluded. The final matched sample included 706 BPCI-A participant hospitals and 1503 matched comparison hospitals.

### Factors and Outcomes

The primary outcome was the change in total episode payments. Total episode payments include payments for care delivered in inpatient, outpatient, and postacute settings, physician services, and durable medical equipment but do not include incentive payments made later by CMS based on program performance. Payments were standardized to account for regional variation and special payments (eg, funding for graduate medical education). Payments were Winsorized at the 1st percentile and 99th percentile according to program specifications and were adjusted to 2019 prices to account for inflation.

Secondary outcomes included changes in patient complexity (the proportion of patients with multimorbidity, frailty, poverty, or the highest-complexity DRG level within each group) and changes in clinical outcomes (90-day readmission, mortality, and healthy days at home, ie, postdischarge days spent outside of a health care setting).^9^ Six or more comorbidities—greater than the median for Medicare beneficiaries—was classified as multimorbidity. Patients in the top quintile of an established claims-based frailty index^10^ were classified as frail. Concurrent enrollment in Medicaid (dual enrollment) was used as a proxy indicator for poverty.

### Statistical Analysis

Patient and hospital characteristics were compared between participating and comparison hospitals overall, for medical episodes, and for surgical episodes. Because of the large sample sizes, comparisons were based on standardized mean differences (SMDs) rather than *P* values, with an SMD greater than 0.10 denoting a statistically significant difference. We used a difference-in-differences approach to determine whether the change from baseline to the intervention period associated with BPCI-A was different for medical conditions compared with surgical conditions. We tested for parallel trends in our primary and secondary outcomes, as a condition for proceeding with difference-in-differences analyses, and ran the standard diagnostic to determine if it was appropriate to complete 3-way testing^11^ (eTable 2 in Supplement 1). For interpretability, total payments were modeled using a linear approach, although generalized estimating equations were used to avoid the normality assumption. In addition, sensitivity analyses were run using a γ distribution and a log-link function. For binary outcomes, a standard logistic model was used with a logit-link function. Each model was run at the episode level and included a match group fixed-effect to control for correlation over time, take advantage of the matched design, and reduce confounding between match groups. The primary predictors were binary indicators for BPCI-A status, period, and medical vs surgical conditions, as well as all 2-way and 3-way interactions. The 3-way interaction term between BPCI-A status, period, and the indicator for medical vs surgical episodes determined whether the effect of BPCI-A participation differed by episode type. In addition to the fixed effects for match groups, all models controlled for age, sex, dual status, disability status, medical comorbidities, and episode complexity based on DRG. We also conducted similar analyses for each individual condition.

Our primary outcome was episode payments and a 2-tailed *P* value less than .05 was considered statistically significant. A number of secondary outcomes were also examined and should be considered exploratory given the large number of outcomes assessed. All analyses were performed from September 2022 to September 2024 using SAS statistical software version 9.4 (SAS Institute) on the Medicare Virtual Research Data Center.

## Results

### Hospital and Episode Characteristics

The final sample included 2 895 878 episodes (Table 1). Of these, 1 618 172 (55.9%) were female, 324 186 (11.2%) under 65 years of age, 1 354 246 (46.8%) between 65 and 80 years, and 1 217 446 (42.0%) older than 80 years. Additionally, 272 114 (9.4%) were Black, 151 307 (5.2%) Hispanic, 2 359 417 (81.5%) White, and 113 040 (3.9%) other race (which included American Indian or Alaska Native, Asian or Pacific Islander, and other). Nonparticipant hospitals had more episodes that qualified for outlier payments from CMS, but cohorts at BPCI-A and comparison hospitals were otherwise similar (eTable 3 in Supplement 1).

**Table 1.** Patient Characteristics

Characteristic	Overall, No. (%)	Medical episodes			Surgical episodes		
		No. (%)		SMD	No. (%)		SMD
		BPCI-A	Non–BPCI-A		BPCI-A	Non–BPCI-A	
Total patients, No.	2 895 878	718 380	1 623 565	NA	147 017	406 916	NA
Patients per episode type per hospital, %	NA	24.7	23.6	−0.044	15.9	17.1	0.044
Age group, y							
<65	324 186 (11.2)	81 804 (11.4)	196 382 (12.1)	0.022	12 640 (8.6)	33 360 (8.2)	−0.014
65-80	1 354 246 (46.8)	305 645 (42.6)	700 874 (43.2)	0.013	90 646 (61.7)	257 081 (63.2)	0.031
>80	1 217 446 (42.0)	330 931 (46.1)	726 309 (44.7)	−0.027	43 731 (29.8)	116 475 (28.6)	−0.025
Female	1 618 172 (55.9)	398 733 (55.5)	893 116 (55.0)	−0.010	87 455 (59.5)	238 868 (58.7)	−0.016
Race or ethnicity							
Black	272 114 (9.4)	78 979 (11.0)	161 715 (10.0)	−0.034	8773 (6.0)	22 647 (5.6)	−0.017
Hispanic	151 307 (5.2)	50 087 (7.0)	78 918 (4.9)	−0.091	7766 (5.3)	14 536 (3.6)	−0.086
White	2 359 417 (81.5)	560 362 (78.0)	1 320 423 (81.3)	0.084	124 445 (84.7)	354 187 (87.0)	0.070
Other^a^	113 040 (3.9)	28 952 (4.0)	62 509 (3.9)	−0.009	6033 (4.1)	15 546 (3.8)	−0.015
Dually enrolled^b^	784 804 (27.1)	214 037 (29.8)	488 833 (30.1)	0.007	22 890 (15.6)	59 044 (14.5)	−0.030
Disability^c^	710 185 (24.5)	178 970 (24.9)	431 050 (26.6)	0.037	27 040 (18.4)	73 125 (18.0)	−0.011
Frail^d^	574 932 (19.9)	166 840 (24.0)	348 374 (22.2)	−0.044	11 443 (8.2)	28 060 (7.2)	−0.036
Multimorbid^e^	1 524 084 (52.6)	422 434 (58.8)	915 520 (56.4)	−0.049	51 053 (34.7)	135 077 (33.2)	−0.032
Comorbidities per patient, mean (SD)	5.85 (3.36)	6.32 (3.43)	6.11 (3.36)	−0.062	4.54 (2.91)	4.45 (2.83)	−0.031
Patients with outlier payments^f^	119 795 (4.1)	8200 (1.1)	82 844 (5.1)	0.187	3301 (2.3)	25 450 (6.3)	0.172

Abbreviations: BPCI-A, Bundled Payments for Care Improvement–Advanced; NA, not applicable; SMD, standardized mean difference.

^a^ This group includes the following categories as reported in Medicare enrollment data: American Indian or Alaska Native, Asian or Pacific Islander, and other.

^b^ Dually enrolled was concurrent enrollment in Medicare and Medicaid.

^c^ Disability was qualified for Medicare via disability.

^d^ Frail was top quintile of frailty index.

^e^ Multimorbid was 6 or more comorbidities.

^f^ Outlier payments were payments from Centers for Medicare & Medicaid Services to hospitals in order to insulate them from losses due to extraordinarily costly episodes.

There were markedly more medical episodes (2 341 945) compared with surgical episodes (553 933), and medical and surgical cohorts were different in several ways (eTable 4 in Supplement 1). Medical episodes were more frequent for patients below 65 years of age and above 80 years of age. There was a higher proportion of White patients in the surgical cohort, and a greater proportion of Black patients in the medical cohort. A greater proportion of medical episodes were for patients dually enrolled in both Medicare and Medicaid, as well as for those who qualified for Medicare via disability. Medical patients had a greater number of comorbidities and were more likely to be frail.

### Changes in Medicare Payments

**Figure 1.**
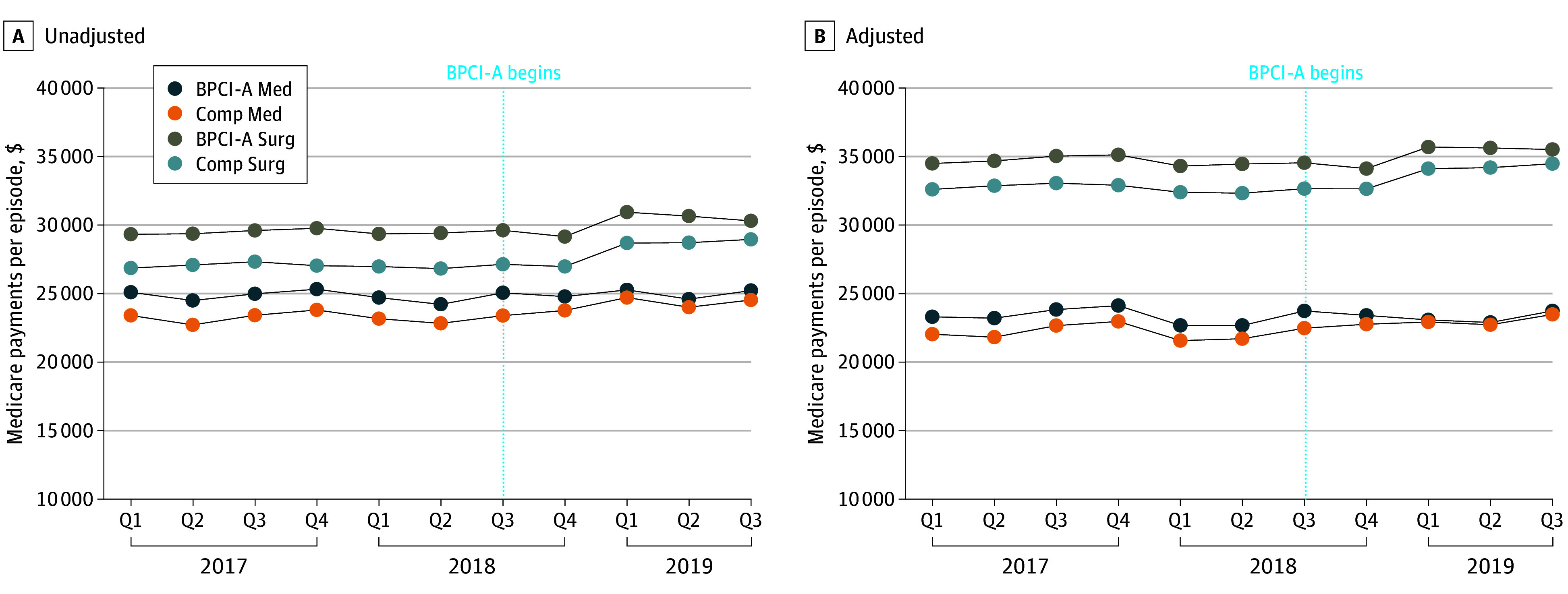
Medicare Payments per Episode for Bundled Payments for Care Improvement–Advanced (BPCI-A) vs Comparison Hospitals BPCI-A Med indicates, medical episode at BPCI-A participating hospital; BPCI-A Surg, surgical episode at BPCI-A participating hospital; Comp Med, medical episode at comparison hospital; Comp Surg, surgical episode at comparison hospital; Q, quarter.

Overall, Medicare payments were higher for surgical than medical episodes, and at BPCI-A vs comparison hospitals, in both unadjusted and adjusted analyses (Figure 1). In the preperiod, the adjusted mean episode payment for medical episodes was $23 345 at BPCI-A hospitals, which declined to $23 269 during the intervention period, for a difference of −$76 per episode (0.33%) (Table 2). Payments at comparison hospitals increased by $806 (3.64%) during the intervention period, resulting in a difference-in-differences (DID) of −$882 (95% CI, −$1004 to −$760). For surgical episodes, baseline payments at BPCI-A hospitals were $34 654 and increased to $35 234, a difference of $579 (1.67%). The increase among comparison hospitals was $1167 per episode (3.57%), yielding a DID of −$587 (95% CI, −$850 to −$324). The triple difference was −$295 (95% CI, −$584 to −$5) (*P* = .05), indicating that the change in payments achieved by BPCI-A participants for medical episodes was similar to that realized in surgical episodes. Patterns were similar for key components of Medicare payments, including index admission and skilled nursing facility (SNF) spending (Table 2; eFigure 1 in Supplement 1). The decrease in readmission payments was significantly greater for medical episodes compared with surgical episodes (−$145 [95% CI, −$273 to −$17]).

**Table 2.** Changes in Episode Payments for BPCI-A and Comparison Hospitals

Medicare payments	$							
	BPCI-A			Nonparticipant			DID (95% CI)	Triple difference (95% CI)
	Pre^a^	Post^a^	Difference	Pre^a^	Post^a^	Difference		
**Total episode**								
Medical	23 345 (23 283 to 23 407)	23 269 (23 186 to 23 352)	−76 (−177 to 25)	22 156 (22 114 to 22 198)	22,962 (22 907 to 23 017)	806 (738 to 874)	−882 (−1004 to −760)	−295 (−584 to −5)
Surgical	34 654 (34 514 to 34 795)	35,234 (35 046 to 35 422)	579 (354 to 805)	32,675 (32 586 to 32 765)	33 842 (33 726 to 33 959)	1167 (1031 to 1302)	−587 (−850 to −324)	
**Index admission**								
Medical	7077 (7065 to 7090)	7935 (7918 to 7952)	857 (836 to 878)	6879 (6870 to 6887)	8129 (8117 to 8140)	1250 (1236 to 1264	−393 (−418 to −368)	−39 (−99 to 21)
Surgical	15 733 (15 704 to 15 763)	16 994 (16 955 to 17 033)	1261 (1214 to 1308)	15 401 (15 383 to 15 420)	17 016 (16 992 to 17 040)	1615 (1587 to 1643)	−354 (−409 to −300)	
**Readmission**								
Medical	4309 (4282 to 4337)	4261 (4224 to 4298)	−48 (−93 to −4)	4187 (4168 to 4205)	4170 (4146 to 4195)	−16 (−46 to 13)	−32 (−86 to 22)	−145 (−273 to −17)
Surgical	3081 (3018 to 3143)	3123 (3040 to 3206)	42 (−57 to 142)	3003 (2964 to 3043)	2933 (2881 to 2984)	71 (−130 to −11)	113 (−3 to 229)	
**Skilled nursing facility**								
Medical	4690 (4660 to 4721)	4168 (4128 to 4209)	−522 (−572 to −472)	4470 (4449 to 4490)	4278 (4251 to 4306	−191 (−225 to −158)	−331 (−391 to −270)	−115 (−258 to 28)
Surgical	8096 (8026 to 8166)	7637 (7544 to 7730)	−459 (−570 to −347)	7575 (7531 to 7619)	7332 (7274 to 7390)	−243 (−310 to −176)	−216 (−346 to −86)	

Abbreviations: BPCI-A, Bundled Payments for Care Improvement–Advanced; DID, difference-in-differences.

^a^ The listed values represent adjusted mean spending during the preprogram period (Q1 2017 to Q3 2018) and the post–BPCI-A period (Q4 2018 to Q3 2019).

In sensitivity analyses using a γ distribution to model cost data, medical episodes had a greater relative decrease in Medicare payments than surgical episodes (rate ratio, 0.962 vs 0.977; ratio of ratios, 0.985; *P* = .003), reflecting similar absolute savings but higher baseline payments in surgical episodes (eTable 5 in Supplement 1). Sensitivity analyses only including the subset of conditions Lewin included in their report yielded similar results; that is, small but statistically significantly higher savings in the medical group (eTable 6 in Supplement 1).

### Clinical Outcomes and Patient Complexity

There were no differential changes in 90-day readmission or mortality associated with BPCI-A participation for medical or surgical episodes (Table 3). BPCI-A participation was associated with a greater increase in healthy days at home for medical (DID, 0.58 [95% CI, 0.43-0.73] days) and surgical episodes (DID, 0.58 [95% CI, 0.26-0.91] days). Fewer patients who were discharged from BPCI-A participant hospitals after admission for a medical diagnosis required SNF admission (DID, –0.4% [95% CI, −0.7% to −0.2%]). For patients who did require SNF admission after hospitalization, BPCI-A participation was associated with a significant decrease in the length of that SNF stay for both medical (−0.52 [95% CI, −0.63 to −0.42] days) and surgical episodes (−0.33 [95% CI, −0.55 to −0.10] days). For each of these outcomes, the triple difference was not significant, suggesting no differential outcome of the BPCI-A program based on episode type.

**Table 3.** Changes in Clinical Outcomes for Patients at BPCI-A and Comparison Hospitals

Outcomes	%							
	BPCI-A			Nonparticipant			DID (95% CI)	Triple difference (95% CI)
	Pre	Post	Difference	Pre	Post	Difference		
**90-d Readmission**								
Medical	30.0 (29.9 to 30.1)	30.0 (29.9 to 30.2)	0.0 (−0.2 to 0.2)	29.7 (29.7 to 29.8)	29.7 (29.5 to 29.8)	−0.1 (−0.2 to 0.1)	0.1 (−0.1 to 0.4)	−0.1 (−0.7 to 0.5)
Surgical	21.9 (21.6 to 22.2)	21.7 (21.3 to 22.1)	−0.2 (−0.7 to 0.3)	21.1 (20.9 to 21.3)	20.7 (20.4 to 20.9)	−0.4 (−0.7 to −0.1	0.2 (−0.3 to 0.8)	
**90-d Mortality**								
Medical	12.1 (12.1 to 12.2)	12.1 (11.9 to 12.2)	−0.1 (−0.2 to 0.1)	12.0 (12.0 to 12.1)	11.9 (11.8 to 12.0)	−0.2 (−0.2 to 0.1)	0.1 (−0.1 to 0.2)	0.2 (−0.2 to 0.7)
Surgical	8.5 (8.3 to 8.7)	8.2 (7.9 to 8.5)	−0.3 (−0.6 to 0.1)	8.4 (8.3 to 8.5)	8.3 (8.1 to 8.5)	−0.1 (−0.3 to 0.1)	−0.2 (−0.6 to 0.2)	
**SNF stay**								
Medical	25.3 (25.1 to 25.4)	24.0 (23.8 to 24.2)	−1.3 (−1.5 to 1.1)	23.8 (23.7 to 23.9)	22.9 (22.8 to 23.0)	−0.9 (−1.0 to −0.7)	−0.4 (−0.7 to −0.2)	−0.3 (−0.9 to 0.3)
Surgical	41.9 (41.6 to 42.2)	40.3 (40.0 to 40.7)	−1.5 (−2.0 to −1.1)	39.0 (38.8 to 39.1)	37.5 (37.3 to 37.8)	−1.4 (−1.7 to −1.2)	−0.1 (−0.6 to 0.4)	
**Length of SNF stay, d**								
Medical	7.78 (7.73 to 7.83)	6.8 (6.74 to 6.88)	−0.97 (−1.05 to -0.88	7.5 (7.44 to 7.51)	7.0 (6.99 to 7.08	−0.44 (−0.50 to −0.38)	−0.52 (−0.63 to −0.42)	−0.19 (−0.44 to 0.44)
Surgical	13.3 (13.2 to 13.4)	12.6 (12.4 to 12.7)	−0.76 (−0.95 to −0.57)	12.5 (12.4 to 12.6)	12.1 (12.0 to 12.2)	−0.44 (−0.55 to −0.32	−0.33 (−0.55 to −0.10)	
**Healthy days at home, d**								
Medical	71.7 (71.6 to 71.8	72.9 (72.8 to 73.0)	1.2 (1.08 to 1.33)	72.4 (72.3 to 72.4)	73 (72.9 to 73.1	0.63 (0.55 to 0.71)	0.58 (0.43 to 0.73)	−0.01 (−0.36 to 0.35)
Surgical	68.6 (68.4 to 68.8)	69.7 (69.5 to 70.0)	1.1 (0.86 to 1.41)	69.9 (69.8 to 70.0)	70.4 (70.3 to 70.6)	0.55 (0.39 to 0.72)	0.58 (0.26 to 0.91)	

Abbreviations: BPCI-A, Bundled Payments for Care Improvement–Advanced; DID, difference-in-differences; SNF, skilled nursing facility.

There was no differential change associated with BPCI-A participation in the proportion of patients with frailty or multimorbidity within the medical or surgical cohorts, or the proportion in the highest DRG category (eTable 7 in Supplement 1). During the program period, both BPCI-A hospitals and nonparticipants cared for a significantly smaller proportion of dual enrollees with medical episodes, but this population decreased to a greater extent at nonparticipant hospitals (DID, 0.25% [95% CI, 0.02%-0.48%]).

### Individual Episodes

**Figure 2.**
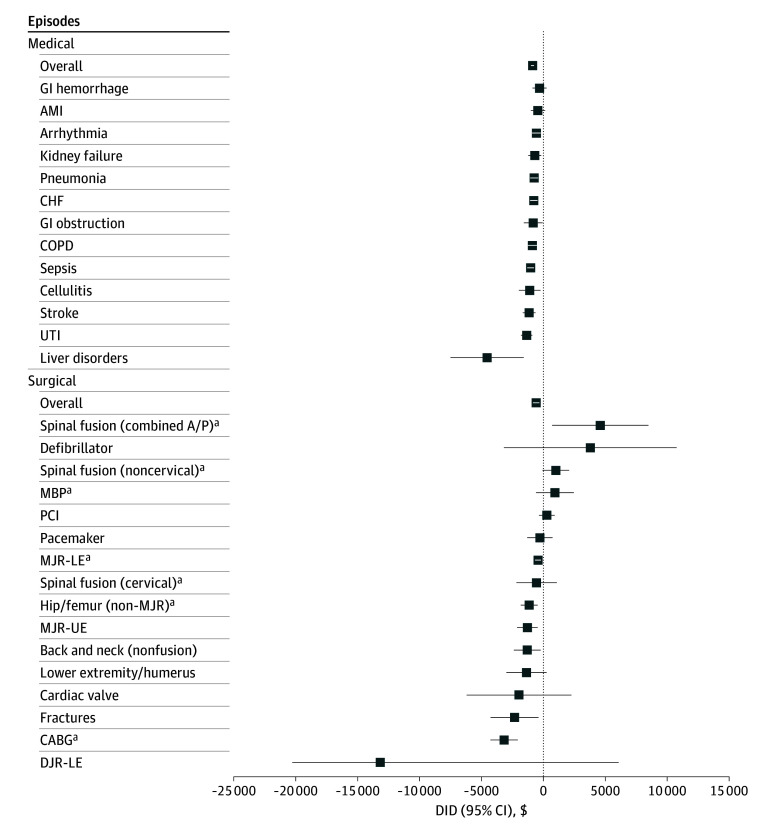
Differences in Medicare Payment Changes for Hospitals With Bundled Payments for Care Improvement–Advanced Participation vs Nonparticipants AMI indicates acute myocardial infarction (n = 219 matched Bundled Payments for Care Improvement–Advanced participating hospitals); arrhythmia, cardiac arrhythmia (n = 277); back and neck, back and neck except spinal fusion (n = 47); CABG, coronary artery bypass graft (n = 62); CHF, congestive heart failure (n = 355); COPD, chronic obstructive pulmonary disease, bronchitis, asthma (n = 229); defibrillator, cardiac defibrillator (n = 6); DJR-LE, double joint replacement of the lower extremity (n = 2); fractures, fractures of the femur and hip or pelvis (n = 43); GI, gastrointestinal; hip/femur (non-MJR), hip and femur procedures except major joint (n = 138); liver disorders, disorders of the liver except malignant neoplasm, cirrhosis, alcoholic hepatitis (n = 34); lower extremity/humerus, lower extremity and/or humerus procedure except hip, foot, femur (n = 67); MBP, major bowel procedure (n = 37); MJR-LE, major joint replacement of the lower extremity (n = 144); MJR-UE, major joint replacement of the upper extremity (n = 37); PCI, percutaneous coronary intervention (n = 72); pneumonia, simple pneumonia and respiratory infections (n = 263); spinal fusion (cervical), cervical spinal fusion (n = 47); spinal fusion (combined A/P), combined anterior posterior spinal fusion (n = 4); UTI, urinary tract infection (n = 227). ^a^Conditions included in Transforming Episode Accountability Model (TEAM) as well.

BPCI-A participation was associated with statistically significant differences in the change in Medicare payments compared with nonparticipants for 11 of 13 medical episodes and 8 of 16 surgical episodes (Figure 2; eTable 8 and eFigure 2 in Supplement 1). However, results should be interpreted with caution given the small sample sizes and wide 95% CIs for several of these episode types, particularly among the surgical episodes.

## Discussion

In this study, BPCI-A participation was associated with significant decreases in Medicare payments for 90-day episodes of care, compared with nonparticipating hospitals. This was true overall, as well as for medical and surgical episodes. While the absolute decreases were similar for medical and surgical episodes, the relative decrease was slightly greater for medical ones. BPCI-A participation was associated with increased healthy days at home as well as decreased length of SNF stay for both medical and surgical episodes. These findings suggest that, broadly, medical and surgical episodes performed similarly within the BPCI-A program.

These findings may have relevance to CMS’s ongoing decision-making around the future of bundled payments, which at present only includes a proposal for surgical episodes. The conditions included in TEAM had a range of performance in the first year of BPCI-A: combined anteroposterior cervical spinal fusion was the only individual condition with a statistically significant increase in spending, while CABG had the greatest statistically significant decrease in spending. It is possible that these differences reflect a greater duration of experience with bundled payments for CABG (which have been bundled to some degree or another since the 1980s), but the precise reasons for differential performance across conditions warrants future investigation.

Previous peer-reviewed literature has shown that BPCI-A participation is associated with modestly decreased episode spending overall,^4^ with similar results in subset analyses of high-risk patients^12^ and 2 medical episodes (sepsis, CHF).^13^ Our findings align with these data and with evaluations of BPCI-A’s predecessor, the Bundled Payments for Care Improvement (BPCI) initiative. Our findings regarding medical vs surgical episodes also agree with a similar prior study of BPCI, which examined all medical and surgical episodes in the program and demonstrated a somewhat higher likelihood of savings in medical than surgical episodes, though there was variability across episode types within these categories.^14^ Studies analyzing BPCI’s impact on spending for individual episode types found no association between participation and payments for some episodes—cardiac procedures,^15^ spinal fusion,^16^ and a collection of common medical diagnoses^17^—but did for joint replacement,^18,19^ and certain medical episodes after several years of participation.^20^ Studies of mandatory bundles for joint replacement also demonstrated success in reducing spending.^6,21,22,23^

Previous studies have consistently reported minimal changes in clinical outcomes associated with participation in BPCI-A or its predecessors.^4,7,12,14,15,17,20,24^ We similarly found no differential changes in mortality or readmission rate associated with BPCI-A participation, but did see significant increases in HDAH for both medical and surgical episodes. The rate of discharge to SNF was significantly decreased across the board, and for patients discharged to a SNF, BPCI-A participation was associated with significantly decreased length of stay for both medical and surgical episodes. The main effect of BPCI-A seems to be in reducing postacute care, perhaps because it is an area with overuse of services, or because reducing postacute payments generally does not reduce hospital revenue; our study does not allow us to tease apart these different mechanisms.

An important contrast to our findings is a program evaluation commissioned by CMS and performed by the Lewin Group, which reported that BPCI-A participation was associated with decreased spending for both medical and surgical episodes in the first 5 quarters of the program, with a greater decrease in payments for surgical episodes.^7^ While these findings differ from ours, there are structural differences in the 2 studies that may explain the discrepancy. The Lewin Group report analyzed 13 total episodes: 10 medical, 2 inpatient surgical, and 1 outpatient percutaneous coronary intervention (PCI). Ours analyzed all 13 medical episodes and all 16 inpatient surgical or procedural episodes. The Lewin Group had stricter inclusion criteria related to previous APM participation at the patient, institution, and market levels. While these restrictions may have resulted in a purer comparison group, it is also an increasingly rare comparison group as participation in some form of APM becomes more common. Our findings should be considered complementary; our study provides an evaluation of BPCI-A’s effectiveness in a current setting and offers pragmatic evidence that can be used to help design future models.

### Limitations

Our study has limitations. The planned duration of the BPCI-A program was 8 years, and we only analyzed the first 12 months. Previous studies have shown that several years of participation may be required before changes in payments and improvements in quality attain statistical significance.^19,24,25^ We only evaluated the hospital program, and not the PGP component; we can draw no conclusions about the efficacy of the PGP experience under bundling. Because we did not have access to PGP data at the episode level, we were unable to exclude these from our hospital episodes, which could bias us toward the null if PGPs were ineffective, or toward a positive result if they were highly effective. Prior reports suggest that PGP and hospital performance were similar for medical episodes and that PGPs performed slightly better than hospitals for surgical ones; PGPs made up a larger proportion of surgical episodes.^7^ The BPCI-A program was paused in 2020 due to the COVID-19 pandemic, but resumed, albeit with a much smaller group of participants; the TEAM model has been proposed to replace BPCI-A in 2026, as aforementioned. There are limitations of the difference-in-differences approach, namely that it relies on the assumption that the trend in the control groups provide a valid counterfactual to the trends in the BPCI-A group. BPCI-A was a voluntary model, and hospitals that chose to participate are different than ones that did not; as such, unmeasured confounders may exist and the average treatment on the treated effects likely would not generalize to nonparticipating hospitals, or to mandatory or broader models.

## Conclusions

In this cohort study analyzing the first year of the BPCI-A program, participation in BPCI-A was associated with similar decreases in Medicare payments for both medical and surgical episodes relative to nonparticipant hospitals. While savings were greater as a proportion of baseline spending for medical conditions, these findings do not support a narrow focus on either episode type as a path forward.

## Notes

Supplement 1. **eTable 1.** Inpatient Episode Types and Volumes Included in BPCI-A and Non-Participant Comparison Group **eTable 2.** Trend Assumptions in a Triple-Difference Design **eTable 3.** Characteristics of Patients at BPCI-A Hospitals and Matched Non-Participant Hospitals **eTable 4.** Characteristics of Medical and Surgical Cohorts **eFigure 1.** Overall Changes in Total and Component Medicare Payments for BPCI-A and Non-Participant Hospitals **eTable 5.** Changes in Payment Under Alternative Specification – Gamma Distribution **eTable 6.** Changes in Payment Under Alternative Specification – Episodes Evaluated in Lewin Report Only **eTable 7.** Changes in Patient Complexity at BPCI-A Hospitals and Non-Participant Hospitals **eTable 8.** Changes in Payments and Clinical Outcomes for Medical and Surgical Episodes at BPCI-A and Comparison Hospitals **eFigure 2.** Quarterly Trends in Unadjusted and Adjusted Medicare Payments for Each Condition in BPCI-A and Comparison Hospitals

Supplement 2.
Data Sharing Statement
