# Supplementary material for: Medical and Surgical Episodes Among Hospital Participants in the Bundled Payments for Care Improvement–Advanced Program
Source: JAMA Netw Open. 2024 Dec 23;7(12):e2451792. doi: 10.1001/jamanetworkopen.2024.51792 (PMC11667361; doi:10.1001/jamanetworkopen.2024.51792)
Supplement: Supplement 1. — eTable 1. Inpatient Episode Types and Volumes Included in BPCI-A and Non-Participant Comparison Group eTable 2. Trend Assumptions in a Triple-Difference Design eTable 3. Characteristics of Patients at BPCI-A Hospitals and Matched Non-Participant Hospitals eTable 4. Characteristics of Medical and Surgical Cohorts eFigure 1. Overall Changes in Total and Component Medicare Payments for BPCI-A and Non-Participant Hospitals eTable 5. Changes in Payment Under Alternative Specification – Gamma Distribution eTable 6. Changes in Payment Under Alternative Specification – Episodes Evaluated in Lewin Report Only eTable 7. Changes in Patient Complexity at BPCI-A Hospitals and Non-Participant Hospitals eTable 8. Changes in Payments and Clinical Outcomes for Medical and Surgical Episodes at BPCI-A and Comparison Hospitals eFigure 2. Quarterly Trends in Unadjusted and Adjusted Medicare Payments for Each Condition in BPCI-A and Comparison Hospitals [file jamanetwopen-e2451792-s001.pdf]

Supplemental Online Content

Robbins KJ, Zheng J, Waken RJ, Orav EJ, Epstein AM, Joynt Maddox KE. Outcomes for medical and surgical episodes among hospital participants in the bundled payments for care improvement advanced program. *JAMA Netw Open.* 2024;7(12):e2451792. doi:10.1001/jamanetworkopen.2024.51792

eTable 1. Inpatient Episode Types and Volumes Included in BPCI-A and Non-Participant Comparison Group

eTable 2. Trend Assumptions in a Triple-Difference Design

eTable 3. Characteristics of Patients at BPCI-A Hospitals and Matched Non-Participant Hospitals

eTable 4. Characteristics of Medical and Surgical Cohorts

eFigure 1. Overall Changes in Total and Component Medicare Payments for BPCI-A and Non-Participant Hospitals

eTable 5. Changes in Payment Under Alternative Specification – Gamma Distribution

eTable 6. Changes in Payment Under Alternative Specification – Episodes Evaluated in Lewin Report Only

eTable 7. Changes in Patient Complexity at BPCI-A Hospitals and Non-Participant Hospitals

eTable 8. Changes in Payments and Clinical Outcomes for Medical and Surgical Episodes at BPCI-A and Comparison Hospitals

eFigure 2. Quarterly Trends in Unadjusted and Adjusted Medicare Payments for Each Condition in BPCI-A and Comparison Hospitals

This supplemental material has been provided by the authors to give readers additional information about their work.

© 2024 Robbins KJ et al. *JAMA Network Open.*

eTable 1. Inpatient episode types and volumes included in BPCI-A and non-participant comparison group.

Condition	BPCI-A (n)	Non-participant (n)	Total (n,%)
Medical	293,880	202,201	496,081 (100%)
Acute myocardial infarction (AMI)	12,191	8,193	20,384 (4.1%)
Cardiac arrhythmia	21,476	14,930	36,406 (7.3%)
Cellulitis	4,578	2,505	7,083 (1.4%)
Congestive heart failure (CHF)	57,330	39,788	97,118 (19.6%)
COPD	23,391	15,590	38,981 (7.9%)
Gastrointestinal hemorrhage	9,766	7,157	16,923 (3.4%)
Gastrointestinal obstruction	4,137	2,827	6,964 (1.4%)
Liver disorders	586	416	1,002 (0.2%)
Pneumonia	31,691	23,374	55,065 (11.1%)
Renal failure	16,694	11,052	27,746 (5.6%)
Sepsis	72,917	50,311	123,228 (24.8%)
Stroke	20,659	13,390	34,049 (6.9%)
Urinary tract infection (UTI)	18,464	12,668	31,132 (6.3%)
Surgical	47,320	34,485	81,805 (100%)
Back / neck (non-fusion)	543	310	853 (1.0%)
Cardiac defibrillator	172	45	217 (0.3%)
Cardiac valve	287	120	407 (0.5%)
Coronary artery bypass graft (CABG)	1,372	846	2,218 (2.7%)
DJR-LE	24	-	24 (0.03%)
Fractures	904	393	1,297 (1.6%)
Hip / femur (non-MJR)	7,696	4,365	12,061 (14.7%)
Lower extremity / humerus	1,662	657	2,319 (2.8%)
Major bowel procedure (MBP)	1,571	899	2,470 (3.0%)
MJR-LE	21,482	18,499	39,981 (48.9%)
MJR-UE	1,260	915	2,175 (2.7%)
Pacemaker	1,886	1,238	3,124 (3.8%)
Percutaneous coronary intervention (PCI)	5,188	4,426	9,614 (11.8%)
Spinal fusion (cervical)	903	511	1,414 (1.7%)
Spinal fusion (combined A/P)	190	19	209 (0.3%)
Spinal fusion (non-cervical)	2,180	1,242	3,422 (4.2%)

Back / neck (non-fusion) = back and neck except spinal fusion. COPD = chronic obstructive pulmonary disease, bronchitis, asthma. DJR-LE = double joint replacement of the lower extremity. Fractures = fractures of the femur and hip or pelvis. Hip / femur (non-MJR) = hip and femur procedures except major joint. Liver disorders = disorders of liver except malignancy, cirrhosis, or alcoholic hepatitis. Lower extremity / humerus = lower extremity and humerus procedure except hip, foot, femur. MJR-LE = major joint replacement of the lower extremity. MJR-UE = major joint replacement of the upper extremity. Pneumonia = simple pneumonia and respiratory infections. Spinal fusion (A/P) = combined anterior posterior spinal fusion.

© 2024 Robbins KJ et al. *JAMA Network Open.*

eTable 2. Trend Assumptions in a Triple-Difference Design

eTable 2a: Trend Assumptions in a Triple-Difference Design[a]: Tests for equal slope differences

Payments / Outcomes	BPCI-A		Non-participant		Difference (p-value)	DID (95% CI)
	Baseline[a]	Slope	Baseline[b]	Slope		
Total episode payments						
Medical	$25,096	-$37	$23,403	-$2	-$36 (0.06)	-$54 (-$140 to $30)
Surgical	$29,323	-$32	$26,863	-$51	$19 (0.63)	
Readmission payments						
Medical	$4,643	$4	$4,406	$13	-$8 (0.32)	-$34 (-$72 to $4)
Surgical	$1,999	$25	$1,899	-$1	$26 (0.14)	
SNF payments						
Medical	$5,411	-$25	$5,106	-$18	-$7 (0.44)	$0.5 (-$42 to $43)
Surgical	$6,055	-$25	$5,375	-$18	-$8 (0.7)	
90-day Readmission Rate (%)						
Medical	32.3	0.02	31.3	0.07	-0.05 (0.23)	-0.0021 (-0.0039 to -0.0004)
Surgical	15.4	0.12	14.5	-0.05	0.17 (0.043)	
90-day Mortality Rate (%)						
Medical	14.5	-0.07	13.6	-0.04	-0.04 (0.17)	-0.0004 (-0.0016 to 0.0009)
Surgical	3.5	-0.06	3.0	-0.06	0.00 (0.99)	
SNF stay (%)						
Medical	28.8	-0.10	26.8	-0.07	-0.00 (0.45)	-0.0003 (-0.002 to 0.0014)
Surgical	33.1	-0.002	29.5	-0.002	0.00 (0.95)	
SNF LOS (days)						
Medical	9.04	-0.04	8.61	-0.03	-0.01 (0.48)	0.0023 (-0.07 to 0.08)
Surgical	9.70	-0.03	8.63	-0.02	-0.01 (0.69)	
HDAH (days)						
Medical	68.7	0.10	70.7	0.05	0.04 (0.07)	0.05 (-0.06 to 0.16)
Surgical	76.0	0.03	77.9	0.04	-0.01 (0.87)	

[a] **Olden, A., Møen, J. (2022). The triple difference estimator. *Econometrics Journal 25*(3), 531–553. https://doi.org/10.1093/ectj/utac010** [b] Baseline = Q1 2017

© 2024 Robbins KJ et al. *JAMA Network Open.*

eTable 2b: Tests for parallel trends by individual conditions

Payments / Outcomes	BPCI-A		Non-participant		Slope Difference (p-value)
	Baseline[a] ($)	Slope($)	Baseline[a] ($)	Slope($)	
**Medical**					
Acute myocardial infarction (AMI)	25,457	-193	23,547	-39	-154(0.09)
Cardiac arrhythmia	18,411	2	17,581	-60	62 (0.25)
Cellulitis	22,902	-315	20,494	-146	-169 (0.19)
Congestive heart failure (CHF)	26,634	-140	24,971	-82	-58 (0.22)
COPD	20,540	-69	18,924	9	-78 (0.14)
Gastrointestinal hemorrhage	20,620	-128	19,519	-84	-44 (0.60)
Gastrointestinal obstruction	17,432	-98	16,014	-20	-78 (0.51)
Liver disorders	30,433	223	26,891	-43	266 (0.57)
Pneumonia	21,598	-72	20,780	-85	13 (0.80)
Renal failure	24,939	7	24,018	-133	140 (0.07)
Sepsis	29,888	-40	27,688	9	-49 (0.24)
Stroke	31,361	-87	30,510	-127	40 (0.62)
Urinary tract infection (UTI)	23,857	-92	22,378	9	-101 (0.14)
**Surgical**					
Back / neck (non-fusion)	14,228	-34	12,642	135	-169 (0.273)
Cardiac defibrillator	44,790	108	50,548	-1,107	1,215 (0.25)
Cardiac valve	54,640	-546	50,701	233	-778 (0.19)
Coronary artery bypass graft (CABG)	41,702	-16	35,786	72	-88 (0.61)
DJR-LE	31,814	1,281	46,244	1,176	105 (0.89)
Fractures	30,032	16	29,823	-140	156 (0.60)
Hip / femur (non-MJR)	43,605	36	42,798	-159	195 (0.07)
Lower extremity / humerus	38,146	-217	35,467	-202	-15 (0.95)
Major bowel procedure (MBP)	29,801	32	29,928	-57	89 (0.70)
MJR-LE	23,691	-174	21,280	-142	-32 (0.37)
MJR-UE	22,913	-207	20,657	-81	-126 (0.34)
Pacemaker	28,507	-92	28,739	-249	157 (0.31)

© 2024 Robbins KJ et al. *JAMA Network Open.*

Payments / Outcomes	BPCI-A		Non-participant		Slope Difference (p-value)
	Baseline[a] ($)	Slope($)	Baseline[a] ($)	Slope($)	
Percutaneous coronary intervention (PCI)	26,106	-108	25,367	-129	21 (0.83)
Spinal fusion (cervical)	27,144	62	25,543	21	41 (0.87)
Spinal fusion (combined A/P)	51,476	-365	47,309	-17	-348 (0.62)
Spinal fusion (non-cervical)	37,483	82	34,667	123	-41 (0.78)

[a] Baseline = Q1 2017

Note: Back / neck (non-fusion) = back and neck except spinal fusion. COPD = chronic obstructive pulmonary disease, bronchitis, asthma. DJR-LE = double joint replacement of the lower extremity. Fractures = fractures of the femur and hip or pelvis. Hip / femur (non-MJR) = hip and femur procedures except major joint. Liver disorders = disorders of liver except malignancy, cirrhosis, or alcoholic hepatitis. Lower extremity / humerus = lower extremity and humerus procedure except hip, foot, femur. MJR-LE = major joint replacement of the lower extremity. MJR-UE = major joint replacement of the upper extremity. Pneumonia = simple pneumonia and respiratory infections. Spinal fusion (A/P) = combined anterior posterior spinal fusion.

© 2024 Robbins KJ et al. *JAMA Network Open.*

eTable 3. Characteristics of patients at BPCI-A hospitals and matched non-participant hospitals.

Variables	All patients (2,895,878)		
	BPCI-A (n,%)	Non-participant (n,%)	SMD
Total hospitals (n)	706	1503	-
Total patients	865,397 (29.9)	2.030.481 (70.1)	-
Patients per episode type per hospital	22.6	21.9	-0.024
Age			
<65	94,444 (10.9)	229,742 (11.3)	0.013
65-79	396,291 (45.8)	957,955 (47.2)	0.028
>80	374,662 (43.3)	842,784 (41.5)	-0.036
Female	486,188 (56.2)	1,131,984 (55.8)	-0.009
Race / ethnicity			
Black	87,752 (10.1)	184,362 (9.1)	-0.036
Hispanic	57,853 (6.7)	93,454 (4.6)	-0.092
White	684,807 (79.1)	1,674,610 (82.5)	0.086
AI/AN, Asian/PI, Other[a]	34,985 (4.0)	78,055 (3.8)	-0.010
Dually enrolled	236,927 (27.4)	547,877 (27.0)	-0.009
Disability	206,010 (23.80)	504,175 (24.8)	0.024
Frail	178,283 (21.4)	376,434 (19.2)	-0.054
Multimorbid	473,487 (54.7)	1,050,597 (51.7)	-0.060
Comorbidities per patient	6.02	5.78	-0.071
Patients with outlier payments	11,501 (1.3)	108,294 (5.3)	0.185

Disability = qualified for Medicare via disability. Dually enrolled = concurrent enrollment in Medicare and Medicaid. Frail = top quintile of frailty index. Multimorbid = six or more comorbidities. Outlier payments = payments from CMS to hospitals in order to insulate them from losses due to extraordinarily costly episodes. SMD = standardized mean difference.

[a] Other includes the following categories: American Indian / Alaska Native, Asian / Pacific Islander, and Other.

© 2024 Robbins KJ et al. *JAMA Network Open.*

eTable 4. Characteristics of medical and surgical cohorts.

Variables	All patients (2,895,878)		
	Medical (n,%)	Surgical (n,%)	SMD
Total patients	2,341,945 (80.9)	553,933 (19.1)	-
Patients per episode type per hospital	24.2	16.8	-0.277
Age			
<65	284,479 (11.9)	46,773 (8.3)	-0.112
65-79	1,029,727 (43.0)	352,583 (62.6)	0.400
>80	1,083,266 (45.2)	163,557 (29.1)	-0.328
Female	1,323,345 (55.2)	331,398 (58.9)	0.074
Race / ethnicity			
Black	246,701 (10.3)	31,773 (5.6)	-0.157
Hispanic	137,093 (5.7)	22,909 (4.1)	-0.072
White	1,918,661 (80.0)	486,273 (86.4)	0.163
AI/AN, Asian/PI, Other[a]	95,017 (4.0)	21,958 (3.9)	-0.003
Dually enrolled	723,169 (30.2)	83,502 (14.8)	-0.341
Disability	623,716 (26.0)	101,781 (18.1)	-0.184
Frail	530,218 (22.9)	40,496 (7.5)	-0.373
Multimorbid	1,373,741 (57.3)	190,137 (33.8)	-0.478
Comorbidities per patient	6.19	4.49	-0.513
Patients with outlier payments	91,784 (3.8)	29,339 (5.2)	0.069

Disability = qualified for Medicare via disability. Dually enrolled = concurrent enrollment in Medicare and Medicaid. Frail = top quintile of frailty index. Multimorbid = six or more comorbidities. Outlier payments = payments from CMS to hospitals in order to insulate them from losses due to extraordinarily costly episodes. SMD = standardized mean difference.

[a] Other includes the following categories: American Indian / Alaska Native, Asian / Pacific Islander, and Other.

© 2024 Robbins KJ et al. *JAMA Network Open.*

eFigure 1. Overall changes in total and component Medicare payments for BPCI-A and non-participant hospitals.

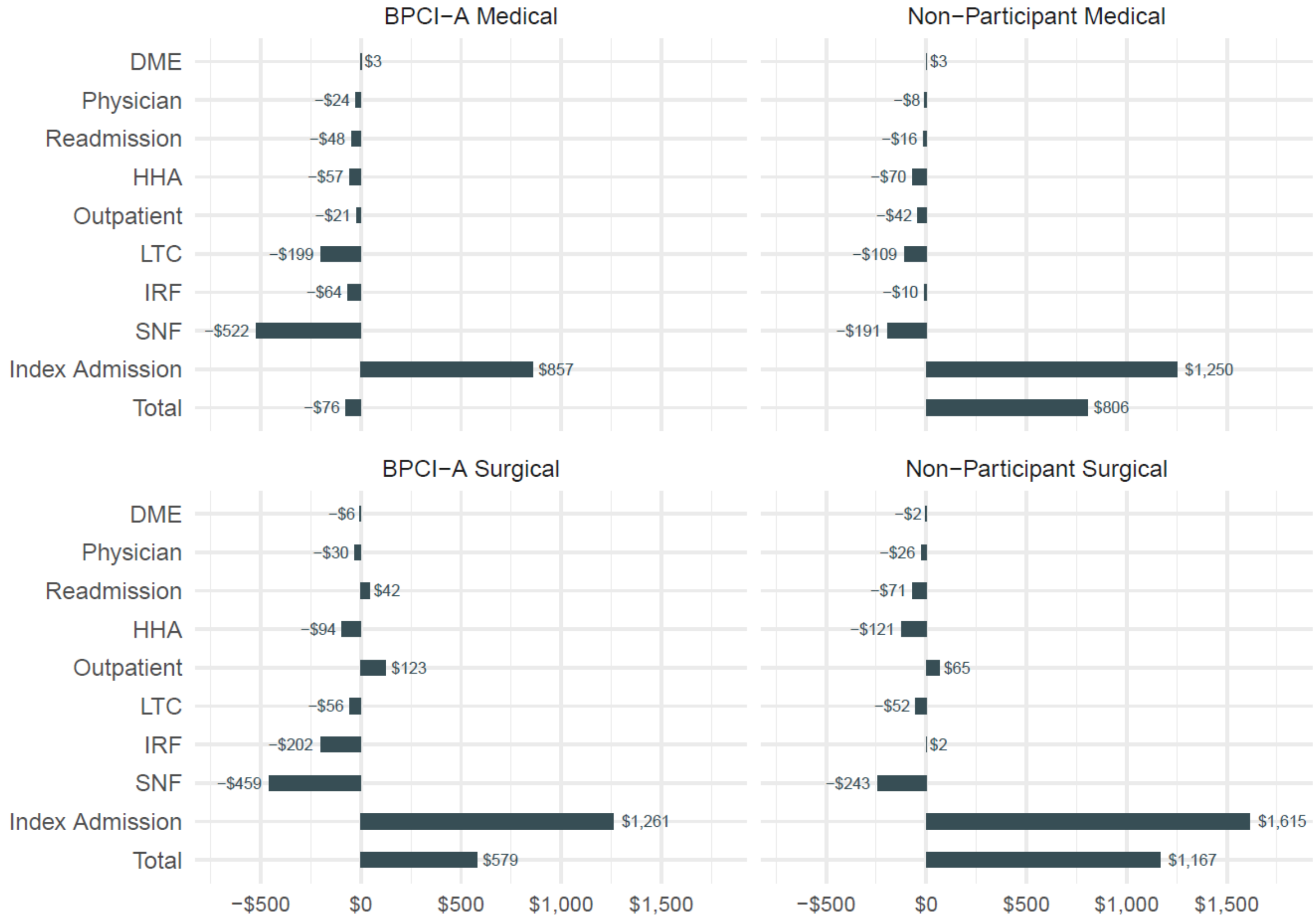

SNF = skilled nursing facility. IRF = inpatient rehabilitation facility. LTC = long-term care. HHA = home health agency. DME = durable medical equipment.

HHA = home health agency. MD = physician payments.

© 2024 Robbins KJ et al. *JAMA Network Open.*

eTable 5. Changes in payment under alternative specification – gamma distribution

Payment	Episode		Pre-Period BPCIA vs. Non	Post-Period BPCIA vs. Non	Diff (Post - Pre) Within BPCI-A or Non BPCI-A	Diff BPCIA vs. Non		3 Way Interaction	
			RR	RR	RR	RR	P	RR	P
Total payment	Medical	BPCI-A	1.049	1.010	0.997	0.962	<.0001	0.985	0.003
		Non-participants	.	.	1.036	.	.	.	.
	Surgical	BPCI-A	1.071	1.046	0.019	0.977	<.0001	.	.
		Non-participants	.	.	0.043	.	.	.	.
SNF payment	Medical	BPCI-A	1.049	0.982	0.90	0.937	<.0001	0.960	0.0004
		Non-participants	.	.	0.97	.	.	.	.
	Surgical	BPCI-A	1.115	1.088	-0.085	0.975	0.0159	.	.
		Non-participants	.	.	-0.06	.	.	.	.
Readmission payment	Medical	BPCI-A	1.014	1.005	0.991	0.991	0.0157	0.953	<.0001
		Non-participants	.	.	1.000	.	.	.	.
	Surgical	BPCI-A	0.996	1.035	0.036	1.040	0.0007	.	.
		Non-participants	.	.	-0.003	.	.	.	.

© 2024 Robbins KJ et al. *JAMA Network Open.*

eTable 6. Changes in payment under alternative specification – episodes evaluated in Lewin Report only.

Payments	BPCI-A			Non-participant			DID (95% CI)
	Pre	Post	Difference	Pre	Post	Difference	
Overall	$25,756	$25,672	-$84	$24,419	$25,153	$734 [a]	-$819 [a] (-$939 to -$698)
Medical	$25,438	$25,328	-$111	$24,164	$24,920	$755 [a]	-$866 [a] (-$1,000 to $732)
Surgical	$27,631	$27,539	-$92	$25,999	$26,546	$547 [a]	-$639 [a] (-$883 to -$396)

[a] = $p < 0.05$

© 2024 Robbins KJ et al. *JAMA Network Open.*

eTable 7. Changes in patient complexity at BPCI-A hospitals and non-participant hospitals.

Variables	BPCI-A			Non-participant			DID (95% CI)	Triple diff (95% CI)
	Pre	Post	Diff	Pre	Post	Diff		
Dually enrolled (%)								
Medical	28.9	28.3	-0.59	28.9	28.0	-0.83	0.25 (0.02 - 0.48)	0.11 (-0.43 to 0.66)
Surgical	21.6	21.3	-0.34	20.7	20.2	-0.47	0.13 (-0.36 to 0.63)	
Frailty (%)								
Medical	20.4	19.9	-0.55	20.1	19.5	-0.61	0.06 (-0.12 to 0.25)	-0.11 (-0.55 to 0.34)
Surgical	19.2	18.8	-0.28	19.4	18.9	-0.46	0.17 (-0.23 to 0.58)	
Multimorbidity (%)								
Medical	52.9	52.8	-0.07	53.1	53.1	0.04	-0.11 (-0.27 to 0.0)	-0.07 (-0.46 to 0.32)
Surgical	51.2	51.5	0.29	50.8	51.1	0.33	-0.04 (-0.39 to 0.32)	
Highest complexity DRG in the family (%)								
Medical	51.8	54	2.13	50.0	52.1	2.05	0.08 (-0.19 to 0.35)	0.18 (-0.46 to 0.81)
Surgical	23.9	24.7	0.83	23.4	24.4	0.93	-0.10 (-0.67 to 0.48)	

Dually enrolled = concurrent enrollment in Medicare and Medicaid. Frail = top quintile of frailty index. Multimorbid = six or more comorbidities.

© 2024 Robbins KJ et al. *JAMA Network Open.*

eTable 8. Changes in payments and clinical outcomes for medical and surgical episodes at BPCI-A and comparison hospitals.

Condition	BPCI-A			Non-participant			DID (95% CI)
	Pre	Post	Difference	Pre	Post	Difference	
Medical episodes							
**AMI**							
Total payments	$24,325	$24,550	$224	$23,223	$23,888	$665	-$441 (-$1019 to $138)
SNF payments	$4,204	$3,646	-$558	$3,996	$3,701	-$296	-$262 (-$509 to -$15)
Readmission (%)	33.5	33.7	0.2	33.4	32.6	-0.7	0.9 (-0.3 to 2.2)
Mortality (%)	15.0	15.0	0.1	15.0	14.8	-0.2	0.3 (-0.6 to 1.2)
HDAH (days)	71.0	72.0	1.00	71.6	72.4	0.82	0.17 (-0.55 to 0.89)
SNF stay (%)	24.2	22.1	-2.1	22.8	21.4	-1.4	-0.7 (-1.8 to 0.4)
SNF LOS (days)	6.91	5.93	-0.98	6.58	5.98	-0.60	-38.6 (-80.7 to 3.4)
**Arrhythmia**							
Total payments	$17,573	$17,596	$23	$17,129	$17,721	$592	-$569 (-$924 to -$214)
SNF payments	$2,727	$2,400	-$326	$2,628	$2,530	-$98	-$228 (-$380 to -$76)
Readmission (%)	27.3	26.7	-0.6	27.5	26.7	-0.7	0.2 (-0.7 to 1.1)
Mortality (%)	6.8	6.6	-0.2	6.9	6.8	-0.2	-0.1 (-0.6 to 0.5)
HDAH (days)	79.6	80.4	0.82	79.7	80.1	0.38	0.44 (0.02 - 0.86)
SNF stay (%)	14.6	13.9	-0.7	14.1	13.6	-0.5	-0.2 (-0.9 to -0.5)

© 2024 Robbins KJ et al. *JAMA Network Open.*

Condition	BPCI-A			Non-participant			DID (95% CI)
	Pre	Post	Difference	Pre	Post	Difference	
SNF LOS (days)	4.32	3.75	-0.57	4.20	3.99	-0.21	-35.7 (-61.7 to -9.6)
**Cellulitis**							
Total payments	$21,607	$21,604	-$3	$20,084	$21,188	$1,104	-$1107 (-$1980 to -$234)
SNF payments	$5,629	$5,142	-$487	$4,996	$5,074	$78	-$565 (-$1055 to -$75)
Readmission (%)	29.8	29.3	-0.5	29.5	29.2	-0.3	-0. (-2.3 to 1.8)
Mortality (%)	5.2	4.7	-0.5	5.5	5.4	-0.1	-0.4 (-1.4 to 0.6)
HDAH (days)	74.7	76.1	1.33	75.8	76.0	0.20	1.12 (0.05 - 2.20)
SNF stay (%)	28.8	26.5	-2.3	24.5	24.6	0.2	-2.3 (-4.2 to -0.5)
SNF LOS (days)	9.57	8.46	-1.11	8.41	8.43	0.02	-1.113 (-197.7 to -28.9)
**COPD**							
Total payments	$19,634	$19,630	-$4	$18,570	$19,453	$883	-$887 (-$1249 to -$529)
SNF payments	$3,235	$2,931	-$304	$3,056	$2,910	-$146	-$158 (-$319 to $2)
Readmission (%)	32.7	33.3	0.6	32.6	32.7	0.2	0.4 (-0.6 to 1.3)
Mortality (%)	7.2	6.7	-0.5	6.9	6.6	-0.4	-0.2 (-0.7 to 0.3)
HDAH (days)	77.6	78.5	0.96	78.1	78.7	0.59	0.37 (-0.06 to 0.81)
SNF stay (%)	18.7	16.8	-1.9	17.1	16.2	-1.0	-1.0 (-1.7 to -0.2)

© 2024 Robbins KJ et al. *JAMA Network Open.*

Condition	BPCI-A			Non-participant			DID (95% CI)
	Pre	Post	Difference	Pre	Post	Difference	
SNF LOS (days)	5.36	4.75	-0.61	5.07	4.74	-0.34	-0.27 (-0.55 to 0.01)
**<u>CHF</u>**							
Total payments	$26,041	$25,328	-$713	$24,832	$24,893	$61	-$774 (-$1077 to -$471)
SNF payments	$4,672	$4,252	-$420	$4,450	$4,183	-$267	-$153 (-$286 to -$20)
Readmission (%)	40.0	39.9	-0.1	39.5	39.4	-0.1	-0.1 (-0.8 to 0.6)
Mortality (%)	15.8	15.1	-0.7	16.1	15.3	-0.8	0.1 (-0.4 to 0.7)
HDAH (days)	69.4	70.9	1.46	69.8	71.0	1.12	0.33 (-0.05 to 0.72)
SNF stay (%)	27.0	25.8	-1.2	25.6	24.3	-1.3	0.1 (-0.5 to 0.7)
SNF LOS (days)	7.81	7.01	-0.79	7.47	6.92	-0.56	-0.24 (-0.47 to 0.08)
**<u>GI hemorrhage</u>**							
Total payments	$19,856	$20,747	$891	$19,174	$20,377	$1,203	-$312 (-$879 to $254)
SNF payments	$3,370	$3,202	-$168	$3,362	$3,313	-$49	-$119 (-$385 to $146)
Readmission (%)	29.2	29.4	0.2	28.8	29.0	0.2	0.0 (-1.4 to 1.4)
Mortality (%)	8.5	8.7	0.2	8.9	8.7	-0.1	0.3 (-0.6 to 1.1)
HDAH (days)	77.1	77.6	0.45	77.1	77.3	0.21	0.24 (-0.46 to 0.95)

© 2024 Robbins KJ et al. *JAMA Network Open.*

Condition	BPCI-A			Non-participant			DID (95% CI)
	Pre	Post	Difference	Pre	Post	Difference	
SNF stay (%)	18.8	18.5	-0.4	17.9	17.9	0.0	-0.3 (-1.5 to 0.8)
SNF LOS (days)	5.50	5.17	-0.33	5.54	5.37	-0.17	-0.15 (-0.61 to 0.30)
**GI obstruction**							
Total payments	$16,135	$15,783	-$353	$15,792	$16,263	$471	-$824 (-$1584 to -$64)
SNF payments	$2,464	$1,992	-$472	$2,374	$2,278	-$96	-$376 (-$706 to -$46)
Readmission (%)	25.9	25.9	0.0	26.9	25.9	-1.0	1.0 (-1.0 to 3.0)
Mortality (%)	6.2	6.2	-0.1	6.7	6.5	-0.1	0.1 (-1.0 to 1.2)
HDAH (days)	80.1	80.9	0.77	79.7	80.2	0.44	0.33 (-0.60 to 1.26)
SNF stay (%)	13.3	11.2	-2.1	12.2	11.7	-0.5	-1.6 (-3.0 to -0.2)
SNF LOS (days)	3.94	3.15	-0.79	3.81	3.56	-0.25	-0.55 (-1.12 to 0.03)
**Liver disorders**							
Total payments	$28,158	$25,650	-$2,508	$25,804	$27,840	$2,036	-$4,545 (-$7,502 to -$1,587)
SNF payments	$4,768	$4,032	-$736	$3,363	$3,337	-$27	-$709 (-$1,821to $402)
Readmission (%)	46.8	42.6	-4.2	44.4	44.7	0.3	-4.5 (-10.9 to 2.0)
Mortality (%)	21.4	20.8	-0.6	21.7	22.2	0.5	-0.0 (-0.06 to 0.04)

© 2024 Robbins KJ et al. *JAMA Network Open.*

Condition	BPCI-A			Non-participant			DID (95% CI)
	Pre	Post	Difference	Pre	Post	Difference	
HDAH (days)	64.9	67.7	2.79	67.5	67.3	-0.22	3.00 (-0.71 to 6.71)
SNF stay (%)	27.1	24.0	-3.1	21.5	21.1	-0.5	-2.6 (-7.8 to 2.7)
SNF LOS (days)	7.90	6.41	-1.49	5.55	5.47	-0.09	-1.40 (-3.29 to 0.49)
**PNA**							
Total payments	$22,108	$21,780	-$328	$21,345	$21,762	$417	-$745 (-$1072 to -$419)
SNF payments	$4,884	$4,266	-$617	$4,690	$4,383	-$307	-$310 (-$477 to -$143)
Readmission (%)	27.4	27.9	0.5	27.7	27.8	0.1	0.4 (-0.4 to 1.2)
Mortality (%)	13.4	13.4	0.1	13.5	13.1	-0.4	0.4 (-0.1 to 1.0)
HDAH (days)	71.3	72.6	1.34	71.6	72.7	1.11	0.24 (-0.21 to 0.68)
SNF stay (%)	27.2	25.7	-1.6	25.9	24.1	-1.9	0.2 (-0.5 to 1.0)
SNF LOS (days)	8.17	7.06	-1.11	7.92	7.27	-0.66	-0.46 (-0.75 to -0.17)
**Renal failure**							
Total payments	$24,438	$24,284	-$154	$23,511	$24,055	$544	-$699 (-$1,219 to -$178)
SNF payments	$6,307	$5,704	-$603	$6,113	$5,790	-$323	-$280 (-$562 to $2)
Readmission (%)	33.7	33.1	-0.6	33.4	33.2	-0.1	-0.5 (-1.6 to 0.7)
Mortality (%)	15.4	14.6	-0.8	15.7	15.9	0.2	-1.0

© 2024 Robbins KJ et al. *JAMA Network Open.*

Condition	BPCI-A			Non-participant			DID (95% CI)
	Pre	Post	Difference	Pre	Post	Difference	
							(-1.9 to -0.1)
HDAH (days)	67.0	68.8	1.78	67.4	67.8	0.49	1.30 (0.59 to 2.00)
SNF stay (%)	32.7	31.5	-1.2	31.1	30.4	-0.7	-0.5 (-1.6 to 0.6)
SNF LOS (days)	10.6	9.51	-1.13	10.4	9.8	-0.62	-0.52 (-1.00 to 0.03)
**Sepsis**							
Total payments	$28,925	$29,120	$194	$27,445	$28,676	$1,231	-$1036 (-$1,312 to -$761)
SNF payments	$6,172	$5,554	-$618	$5,797	$5,600	-$198	-$420 (-$552 to -$288)
Readmission (%)	30.9	30.9	0.0	30.7	30.7	0.0	0.0 (-0.6 to 0.5)
Mortality (%)	18.2	18.0	-0.2	17.2	17.1	-0.1	-0.1 (-0.5 to 0.4)
HDAH (days)	64.4	66.0	1.54	66.0	66.7	0.73	0.81 (0.47 - 1.15)
SNF stay (%)	33.3	31.6	-1.7	30.8	29.7	-1.1	-0.6 (-1.2 to -0.1)
SNF LOS (days)	10.5	9.30	-1.19	9.93	9.40	-0.53	-0.66 (-0.89 to -0.43)
**Stroke**							
Total payments	$30, 548	$29,711	-$837	$29,820	$30,133	$313	-$1,150 (-$1,664 to -$635)
SNF payments	$7,365	$6,389	-$975	$7,796	$7,253	-$543	-$432 (-$706 to -$159)
Readmission (%)	24.0	24.0	-0.0	24.0	23.3	-0.7	0.7 (-0.2 to 1.5)
Mortality (%)	14.0	14.6	0.6	14.1	14.2	0.1	0.5

© 2024 Robbins KJ et al. *JAMA Network Open.*

Condition	BPCI-A			Non-participant			DID (95% CI)
	Pre	Post	Difference	Pre	Post	Difference	
							(-0.1 to 1.2)
HDAH (days)	62.7	64.2	1.56	62.3	63.4	1.21	0.44 (-0.16 to 1.04)
SNF stay (%)	31.2	29.9	-1.4	32.5	31.3	-1.2	-0.1 (-1.0 to 0.8)
SNF LOS (days)	11.9	10.3	-1.74	12.7	11.8	-0.95	-0.67 (-1.14 to -0.23)
**UTI**							
Total payments	$23,139	$22,976	-$164	$22,167	$23,356	$1,188	-$1,352 (-$1,808 to -$896)
SNF payments	$7,684	$6,980	-$705	$7,472	$7,577	$105	-$810 (-$1,103 to -$516)
Readmission (%)	30.8	30.8	0.0	30.5	31.0	0.5	-0.5 (-1.5 to 0.6)
Mortality (%)	9.5	9.8	0.2	10.1	10.2	0.1	0.1 (-0.6 to 0.8)
HDAH (days)	69.2	70.5	1.30	69.1	69.0	-0.08	1.38 (0.74 to 2.01)
SNF stay (%)	38.0	37.3	-0.7	36.5	37.1	0.6	-1.3 (-2.4 to -0.2)
SNF LOS (days)	12.9	11.6	-1.24	12.8	12.9	0.04	-1.32 (-1.82 to -0.82)
Surgical episodes							
**Back / neck (non-fusion)**							
Total payments	$13,243	$14,444	$1,202	$13,157	$15,666	$2,509	-1,307 (-2,387 to -228)
SNF payments	$3,216	$3,402	$187	$3,518	$3,778	$260	-$73 (-$987 to $841)

© 2024 Robbins KJ et al. *JAMA Network Open.*

Condition	BPCI-A			Non-participant			DID (95% CI)
	Pre	Post	Difference	Pre	Post	Difference	
Readmission (%)	-	-	-	-	-	-	-
Mortality (%)	1.4	1.5	0.1	1.1	1.6	0.5	-0.4 (-1.6 to 0.8)
HDAH (days)	84.2	84.1	-0.06	84.0	83.4	-0.62	0.57 (-1.05 to 2.19)
SNF stay (%)	18.4	19.8	1.4	19.8	20.9	1.1	0.3 (-3.7 to 4.3)
SNF LOS (days)	5.14	5.21	0.08	5.47	5.81	0.34	-0.27 (-1.76 to 1.23)
**CABG**							
Total payments	$39,734	$41,995	$2,261	$36,373	$41,803	$5,430	-$3170 (-$4272 to -$2067)
SNF payments	$3,012	$2,442	-$570	$2,918	$2,559	-$358	-$212 (-$567 to $143)
Readmission (%)	20.7	19.9	-0.8	19.3	18.8	-0.5	-0.3 (-2.4 to 1.7)
Mortality (%)	1.7	1.5	-0.2	1.6	1.4	-0.2	0.0 (-0.6 to 0.7)
HDAH (days)	80.6	82.1	1.57	81.6	82.3	0.72	0.85 (0.03 to 1.67)
SNF stay (%)	20.8	18.1	-2.8	21.8	19.4	-2.4	-0.4 (-2.4 to 1.6)
SNF LOS (days)	4.71	3.63	-1.07	4.54	3.89	-0.65	-0.42 (-0.99 to 0.16)
**Cardiac valve**							
Total payments	$52,858	$49,818	-$3,039	$50,022	$48,952	-$1,070	-$1,970 (-$6,199 to $2,260)
SNF payments	$3,524	$2,667	-$857	$3,210	$3,253	$43	-$900

© 2024 Robbins KJ et al. *JAMA Network Open.*

Condition	BPCI-A			Non-participant			DID (95% CI)
	**Pre**	**Post**	**Difference**	**Pre**	**Post**	**Difference**	
							(-$2,427 to $627)
Readmission (%)	24.1	26.5	2.4	24.1	23.9	-0.2	2.6 (-5.6 to 10.7)
Mortality (%)	1.9	2.8	0.9	2.7	3.6	1.0	-0.1 (-3.2 to 3.1)
HDAH (days)	78.5	80.9	2.38	80.3	79.1	-1.16	3.53 (-0.10 to 7.05)
SNF stay (%)	22.4	20.3	-2.1	20.0	20.6	0.6	-2.6 (-10.0 to 4.7)
SNF LOS (days)	5.63	4.14	-1.49	4.45	4.62	0.18	-1.67 (-3.950 to 0.62)
**Defibrillator**							
Total payments	$53,494	$52,921	-$574	$46,518	$42,157	-$4,361	$3,788 (-$3,187 to $10,762)
SNF payments	$2,057	$1,891	-$166	$2,064	$1,945	-$119	-$47 (-$1,882 to $1,787)
Readmission (%)	35.3	34.5	-0.7	27.5	33.0	5.5	-6.2 (-20.8 to 8.4)
Mortality (%)	6.8	4.1	-2.8	2.9	4.7	1.7	-4.5 (-11.0 to 2.0)
HDAH (days)	79.3	79.8	0.48	82.9	82.8	-0.01	0.49 (-4.89, 5.87)
SNF stay (%)	15.3	13.2	-2.1	11.9	12.9	1.0	-3.1 (-13.6 to 7.4)
SNF LOS (days)	2.91	2.98	0.07	2.95	3.23	0.28	-0.21 (-3.1 to 2.77)

© 2024 Robbins KJ et al. *JAMA Network Open.*

Condition	BPCI-A			Non-participant			DID (95% CI)
	Pre	Post	Difference	Pre	Post	Difference	
**DJR-LE**							
Total payments	$36,481	$37,292	$812	$48,344	$62,333	$13,989	-$13,177 (-$20,276 to -$6,079)
SNF payments	$1,638	$4,469	$2,831	$3,874	$3,794	-$80	$2,911 (-$1,089 to $6,912)
Readmission (%)	4.6	19.9	15.3	9.6	6.9	-2.8	18.1 (0.7 - 35.5)
Mortality (%)	-	-	-	-	-	-	-
HDAH (days)	82.7	76.2	-6.46	74.8	73.1	-1.76	-4.70 (-11.14 to 1.74)
SNF stay (%)	18.9	28.3	9.4	5.3	26.3	21.1	-11.7 (-42.8 to 19.5)
SNF LOS (days)	2.63	8.10	5.46	6.02	6.71	0.69	4.78 (-1.85 to 11.4)
**Fractures**							
Total payments	$30,379	$28,601	-$1,777	$29,454	$30,008	$554	-$2,331 (-$4,267 to -$395)
SNF payments	$14,605	$12,448	-$2,157	$14,786	$14,254	-$532	-$1,625 (-$3,054 to -$196)
Readmission (%)	22.8	23.3	0.5	21.7	21.0	-0.6	1.1 (-2.9 to 5.2)
Mortality (%)	14.6	14.3	-0.3	14.4	12.6	-1.8	1.5 (-1.8 to 4.7)
HDAH (days)	53.0	57.4	4.35	53.2	55.2	2.03	2.32 (-0.47 to 5.10)
SNF stay (%)	66.9	63.3	-3.6	68.7	69.1	0.4	-4.0 (-8.5 to 0.5)

© 2024 Robbins KJ et al. *JAMA Network Open.*

Condition	BPCI-A			Non-participant			DID (95% CI)
	Pre	Post	Difference	Pre	Post	Difference	
SNF LOS (days)	24.5	20.8	-3.69	24.9	23.9	-1.02	-2.67 (-5.06 to -0.27)
**Hip / femur**							
Total payments	$43,583	$42,408	-$1,174	$42,651	$42,624	-$28	-$1147 (-$1829 to -$465)
SNF payments	$17,500	$16,082	-$1,418	$18,292	$17,340	-$952	-$467 (-$1000 to $66)
Readmission (%)	22.1	21.8	-0.3	21.6	20.5	-1.1	0.8 (-0.5 to 2.2)
Mortality (%)	9.9	9.4	-0.6	9.8	10.1	0.4	-1.0 (-1.90 to 0.0)
HDAH (days)	51.1	54.6	3.51	50.3	52.3	1.95	1.57 (0.66 - 2.47)
SNF stay (%)	72.1	73.5	1.4	73.9	73.2	-0.7	2.1 (0.7 - 3.5)
SNF LOS (days)	28.6	26.3	-2.34	30.3	28.6	-1.65	-0.69 (-1.57 to 0.19)
**Lower ext / humerus**							
Total payments	$37,987	$37,728	-$259	$36,083	$37,185	$1,102	-$1,361 (-$2,987 to $264)
SNF payments	$13,702	$12,819	-$883	$13,019	$12,551	-$467	-$415 (-$1,564 to $733)
Readmission (%)	22.0	18.6	-3.5	20.5	21.4	0.9	-4.4 (-7.4 to -1.4)
Mortality (%)	3.3	2.4	-0.9	3.4	3.4	0.1	-1.0 (-2.3 to 0.4)
HDAH (days)	62.0	64.1	2.14	63.0	63.9	0.82	1.31 (-0.37 to 3.36)

© 2024 Robbins KJ et al. *JAMA Network Open.*

Condition	BPCI-A			Non-participant			DID (95% CI)
	Pre	Post	Difference	Pre	Post	Difference	
SNF stay (%)	56.8	55.6	-1.2	53.6	53.6	0.1	-1.3 (-4.7 to 2.2)
SNF LOS (days)	22.9	21.3	-1.62	21.9	21.1	-0.85	-0.77 (-2.70 to 1.17)
**MBP**							
Total payments	$30,152	$33,196	$3,044	$29,352	$31,467	$2,115	$929 (-$593 to $2,451)
SNF payments	$3,357	$3,129	-$228	$3,612	$3,194	-$418	$190 (-$411 to $791)
Readmission (%)	26.0	25.8	-0.2	25.3	23.8	-1.5	1.3 (-1.8 to 4.4)
Mortality (%)	5.2	4.7	-0.5	5.2	5.0	-0.1	-0.3 (-1.9 to 1.2)
HDAH (days)	78.5	79.3	0.88	78.4	79.6	1.20	-0.32 (-1.76 to 1.13)
SNF stay (%)	21.4	20.2	-1.2	20.6	18.0	-2.6	1.4 (-1.3 to 4.0)
SNF LOS (days)	5.28	5.00	-0.28	5.64	4.78	-0.86	0.59 (-0.38 to 1.54)
**MJR-LE**							
Total payments	$23,383	$23,587	$203	$21,615	$22,256	$642	-$438 (-$681 to -$195)
SNF payments	$4,228	$3,656	-$572	$3,716	$3,432	-$284	-$288 (-$425 to -$151)
Readmission (%)	11.1	11.1	0.0	10.5	9.9	-0.6	0.6 (0.0 to 1.1)
Mortality (%)	1.5	1.5	-0.1	1.4	1.4	0.0	-0.1 (-0.3 to 0.1)
HDAH (days)	81.0	82.1	1.06	82.2	82.7	0.50	0.56

© 2024 Robbins KJ et al. *JAMA Network Open.*

Condition	BPCI-A			Non-participant			DID (95% CI)
	Pre	Post	Difference	Pre	Post	Difference	
							(0.29 to 0.82)
SNF stay (%)	29.5	26.2	-3.4	26.0	23.5	-2.6	-0.8 (-1.5 to -0.1)
SNF LOS (days)	6.57	5.66	-0.91	5.80	5.31	-0.49	-0.42 (-0.64 to -0.20)
**MJR-UE**							
Total payments	$22,453	$22,584	$131	$21,084	$22,507	$1,422	-$1,292 (-$2,119 to -$464)
SNF payments	$2,549	$1,998	-$550	$2,306	$1,911	-$395	-$155 (-$646 to $335)
Readmission (%)	8.5	7.1	-1.4	8.0	7.7	-0.3	-1.1 (-3.0 to 0.9)
Mortality (%)	0.4	0.7	0.3	0.4	0.6	0.2	0.1 (-0.4 to 0.6)
HDAH (days)	85.3	86.2	0.91	85.7	86.4	0.62	0.28 (-0.63 to 1.20)
SNF stay (%)	13.7	11.5	-2.2	12.7	11.0	-1.7	-0.5 (-2.7 to 1.8)
SNF LOS (days)	3.84	2.91	-0.93	3.48	2.81	-0.67	-0.26 (-1.07 to 0.56)
**Pacemaker**							
Total payments	$28,034	$28,843	$809	$27,661	$28,756	$1,095	-$286 (-$1,308 to $736)
SNF payments	$3,657	$3,620	-$37	$3,692	$3,425	-$267	$230 (-$276 to $735)
Readmission (%)	20.8	20.5	-0.3	21.2	20.8	-0.4	0.1 (-2.3 to 2.5)
Mortality (%)	4.0	4.4	0.3	4.3	3.9	-0.3	0.7 (-0.5 to 1.9)

© 2024 Robbins KJ et al. *JAMA Network Open.*

Condition	BPCI-A			Non-participant			DID (95% CI)
	Pre	Post	Difference	Pre	Post	Difference	
HDAH (days)	79.9	80.1	0.17	80.0	80.5	0.49	-0.32 (-1.46 to 0.82)
SNF stay (%)	21.9	22.4	0.5	19.9	19.1	-0.9	1.4 (-0.8 to 3.6)
SNF LOS (days)	5.9	5.7	-0.13	5.8	5.5	-0.35	0.22 (-0.62 to 1.06)
**PCI**							
Total payments	$24,985	$26,103	$1,118	$24,543	$25,383	$840	$277 (-$362 to $916)
SNF payments	$1,454	$1,430	-$24	$1,405	$1,340	-$65	$41 (-$165 to $247)
Readmission (%)	22.0	21.1	-0.9	21.7	21.8	0.1	-1.0 (-2.6 to 0.7)
Mortality (%)	3.5	3.3	-0.2	3.0	2.9	-0.1	-0.1 (-0.8 to 0.6)
HDAH (days)	84.0	84.4	0.41	84.5	84.6	0.15	0.25 (-0.35 to 0.86)
SNF stay (%)	8.4	7.5	-0.9	7.6	7.3	-0.3	-0.6 (-1.6 to 0.4)
SNF LOS (days)	2.13	2.04	-0.07	2.04	1.90	-0.14	0.07 (-0.29 to 0.42)
**Spinal fusion (A/P)**							
Total payments	$46,673	$45,552	-$1,151	$47,863	$42,119	-$5,743	$4,592 ($704 to $8,481)
SNF payments	$4,177	$2,640	-$1,537	$2,986	$2,581	-$405	-$1,132 (-$2,608 to $344)
Readmission (%)	13.5	9.8	-3.7	13.1	13.2	0.1	-3.8 (-10.9 to 3.2)

© 2024 Robbins KJ et al. *JAMA Network Open.*

Condition	BPCI-A			Non-participant			DID (95% CI)
	Pre	Post	Difference	Pre	Post	Difference	
Mortality (%)	0.0	1.7	1.7	0.8	0.5	-0.2	1.9 (0.1 to 3.6)
HDAH (days)	81.3	83.8	2.54	82.0	82.8	0.81	1.73 (-1.42 to 4.88)
SNF stay (%)	28.6	21.3	-7.3	24.0	17.9	-6.1	-1.2 (-9.6 to 7.3)
SNF LOS (days)	6.77	4.20	-2.56	4.78	3.95	-0.83	-1.73 (-4.14 to 0.68)
**Spinal fusion (cervical)**							
Total payments	$29,958	$31,020	$1,063	$28,027	$29,639	$1,612	-$549 (-$2,177 to $1,078)
SNF payments	$2,788	$2,780	-$9	$3,154	$2,634	-$520	$511 (-$164 to $1,187)
Readmission (%)	14.8	15.2	0.5	13.9	11.9	-2.1	2.5 (-0.4 to 5.3)
Mortality (%)	1.5	0.9	-0.6	1.5	1.4	-0.1	-0.5 (-1.5 to 0.5)
HDAH (days)	81.3	82.0	0.69	81.4	82.5	1.06	-0.37 (-1.80 to 1.05)
SNF stay (%)	14.8	15.7	0.9	15.4	12.8	-2.7	3.6 (0.8 to 6.4)
SNF LOS (days)	4.23	4.18	-0.05	4.86	3.98	-0.89	0.84 (-0.27 to 1.94)
**Spinal fusion (non-cervical)**							
Total payments	$38,595	$41,021	$2,426	$35,701	$37,124	$1,423	$1,004 (-$70 to $2,077)
SNF payments	$3,495	$3,235	-$260	$3,147	$2,810	-$337	$77 (-$341 to $494)

© 2024 Robbins KJ et al. *JAMA Network Open.*

Condition	BPCI-A			Non-participant			DID (95% CI)
	Pre	Post	Difference	Pre	Post	Difference	
Readmission (%)	14.1	14.9	0.8	13.9	12.6	-1.2	2.1 (0.1 to 4.0)
Mortality (%)	0.8	1.0	0.2	0.7	0.7	0.0	0.1 (-0.4 to 0.6)
HDAH (days)	80.8	80.9	0.13	82.0	82.7	0.72	-0.59 (-1.48 to 0.31)
SNF stay (%)	24.8	22.0	-2.9	21.2	19.8	-1.4	-1.5 (-3.7 to 0.7)
SNF LOS (days)	5.55	5.22	-0.33	4.93	4.41	-0.52	0.20 (-0.50 to 0.89)

AMI = acute myocardial infarction. Arrhythmia = cardiac arrhythmia. Back / neck (non-fusion) = back and neck except spinal fusion. CABG = coronary artery bypass graft. CHF = congestive heart failure. COPD = chronic obstructive pulmonary disease, bronchitis, asthma. Defibrillator = cardiac defibrillator. DJR-LE = double joint replacement of the lower extremity. Fractures = fractures of the femur and hip or pelvis. GI hemorrhage = gastrointestinal hemorrhage. GI obstruction = gastrointestinal obstruction. HDAH = healthy days at home. Hip / femur (non-MJR) = hip and femur procedures except major joint. Liver disorders = disorders of liver except malignancy, cirrhosis, or alcoholic hepatitis.. Lower ext / humerus = lower extremity and humerus procedure except hip, foot, femur. MBP = major bowel procedure. MJR-LE = major joint replacement of the lower extremity. MJR-UE = major joint replacement of the upper extremity. PCI = percutaneous coronary intervention (inpatient). PNA = simple pneumonia and respiratory infections. SNF LOS = mean length of SNF stay for those discharged to SNF. SNF stay = percentage of patients with SNF stay after discharge. Spinal fusion (A/P) = combined anterior posterior spinal fusion. UTI = urinary tract infection.

© 2024 Robbins KJ et al. *JAMA Network Open.*

eFigure 2. Quarterly trends in unadjusted and adjusted Medicare payments for each condition in BPCI-A and comparison hospitals.

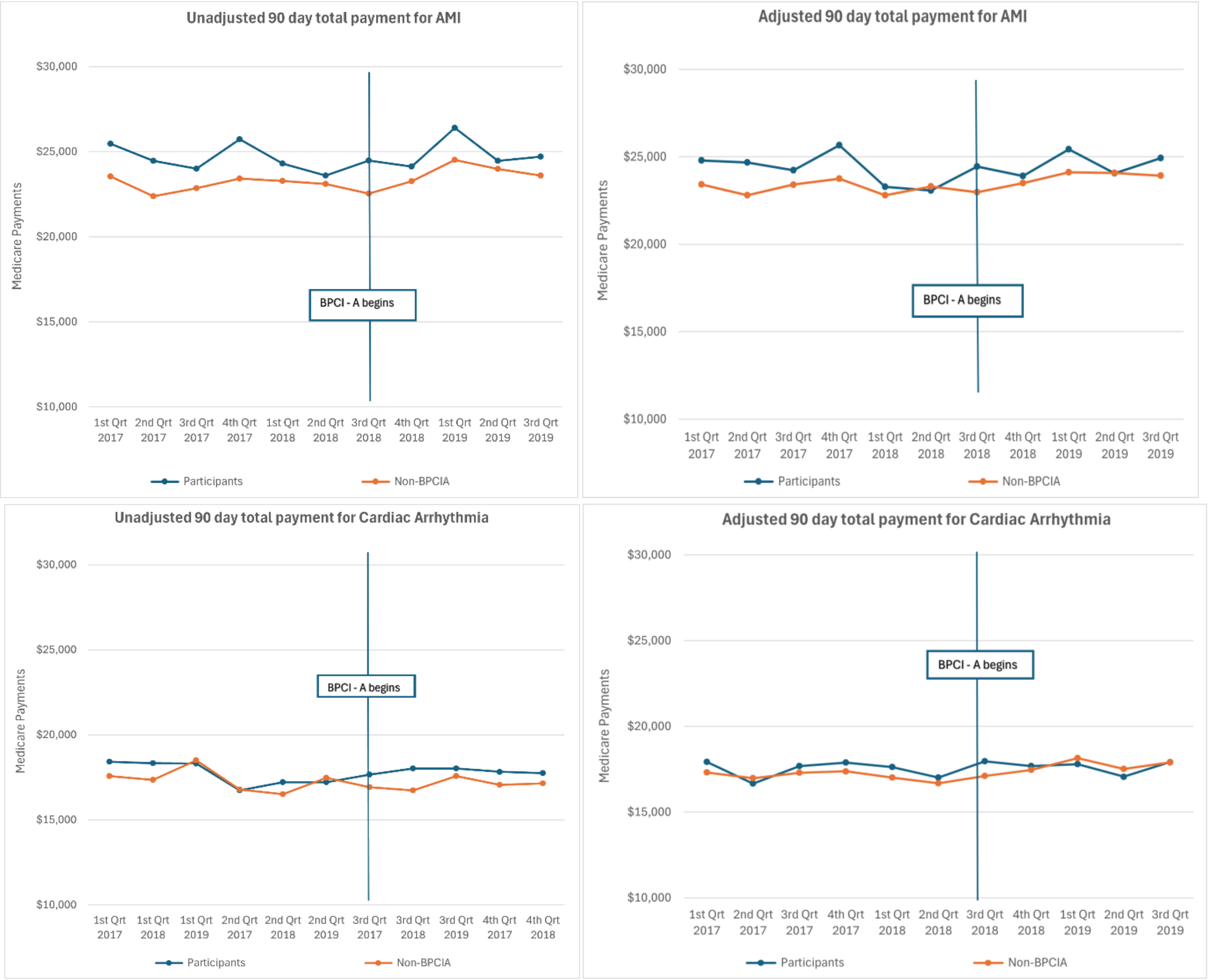

© 2024 Robbins KJ et al. *JAMA Network Open.*

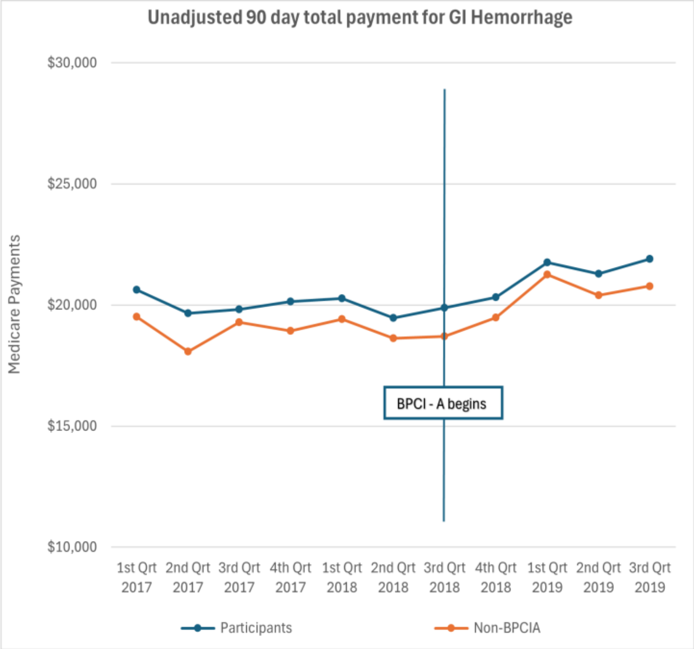

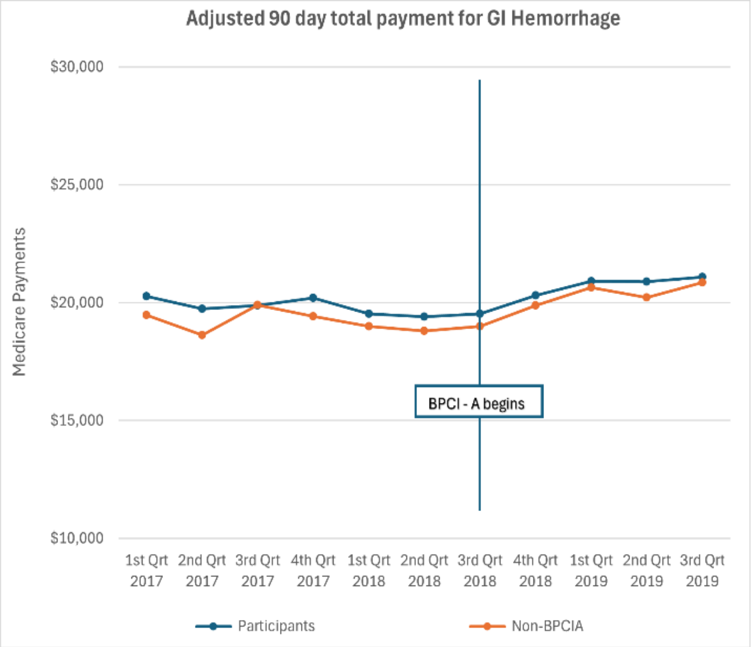

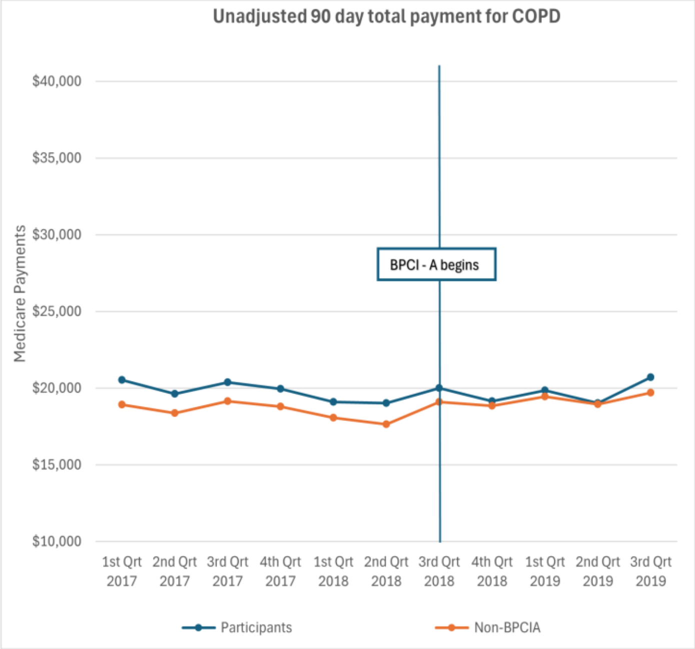

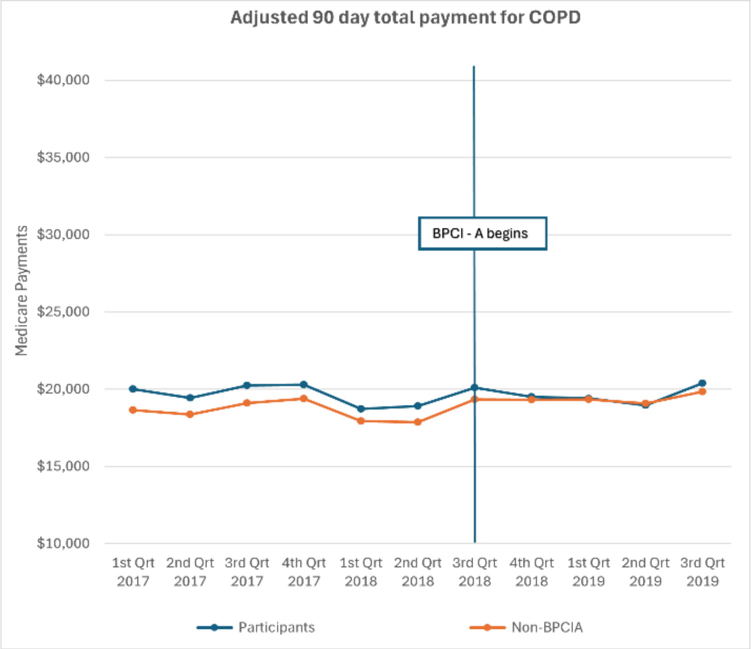

© 2024 Robbins KJ et al. *JAMA Network Open.*

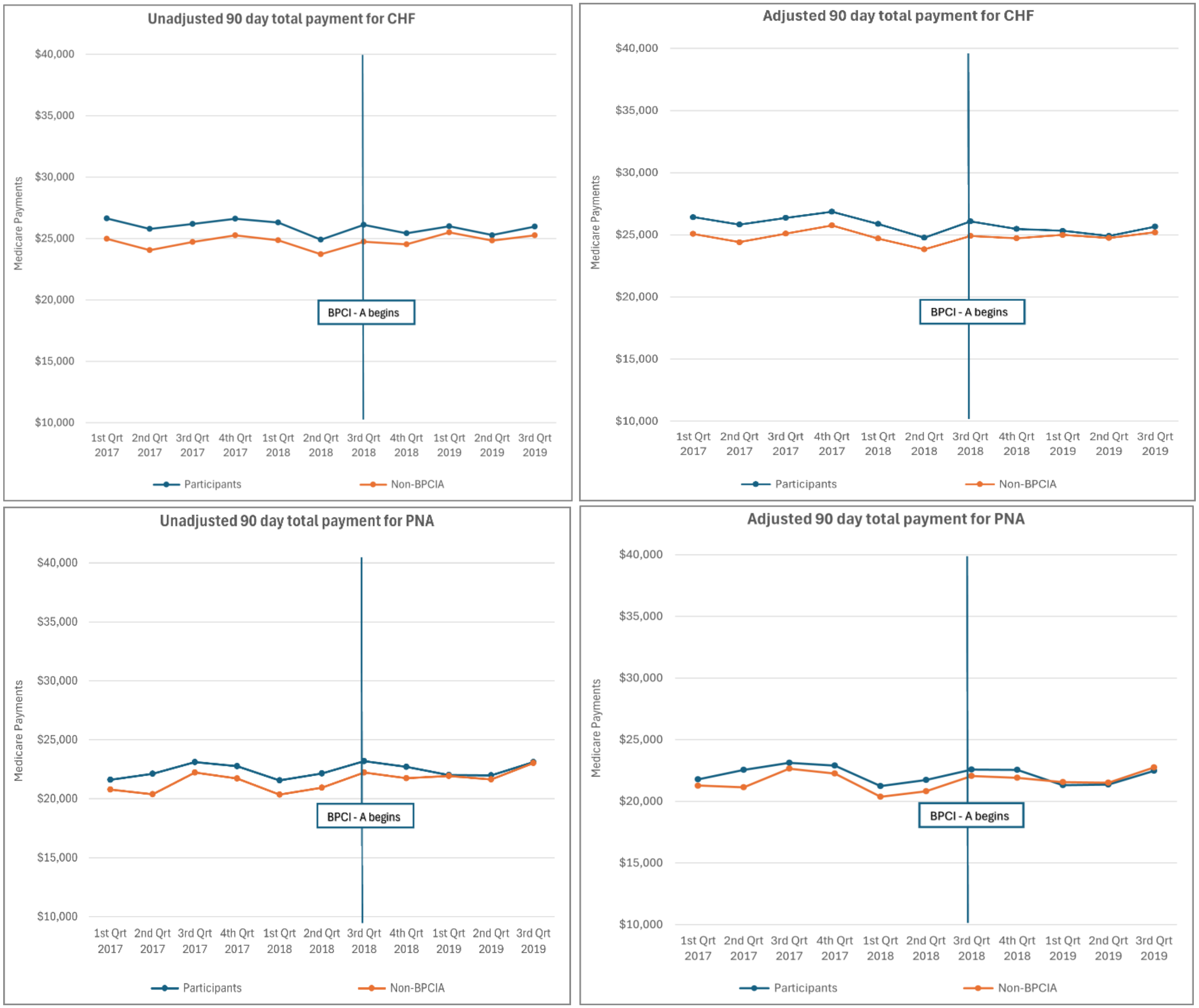

© 2024 Robbins KJ et al. *JAMA Network Open.*

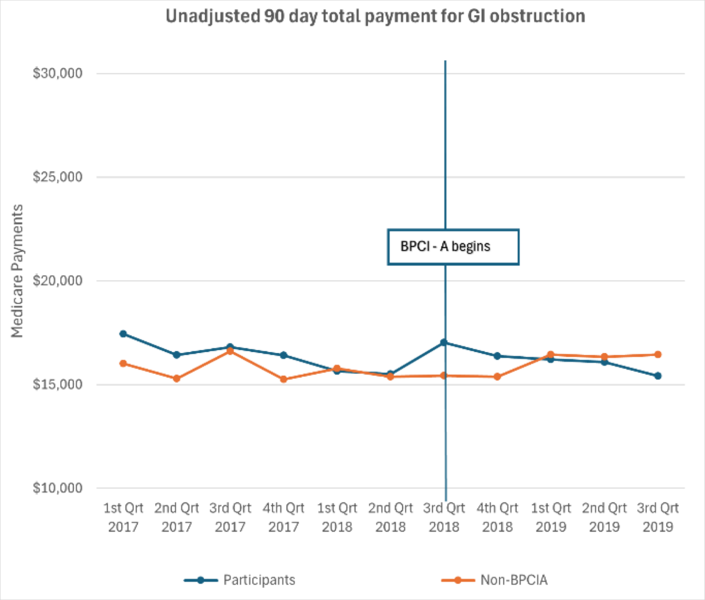

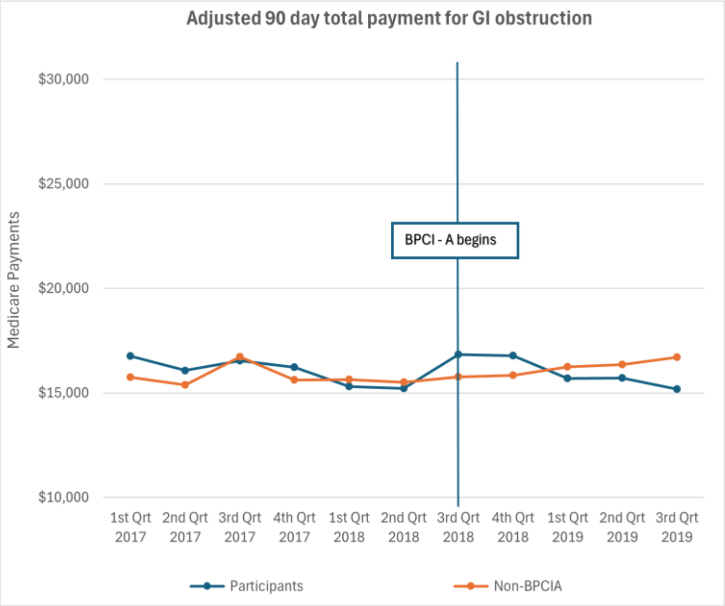

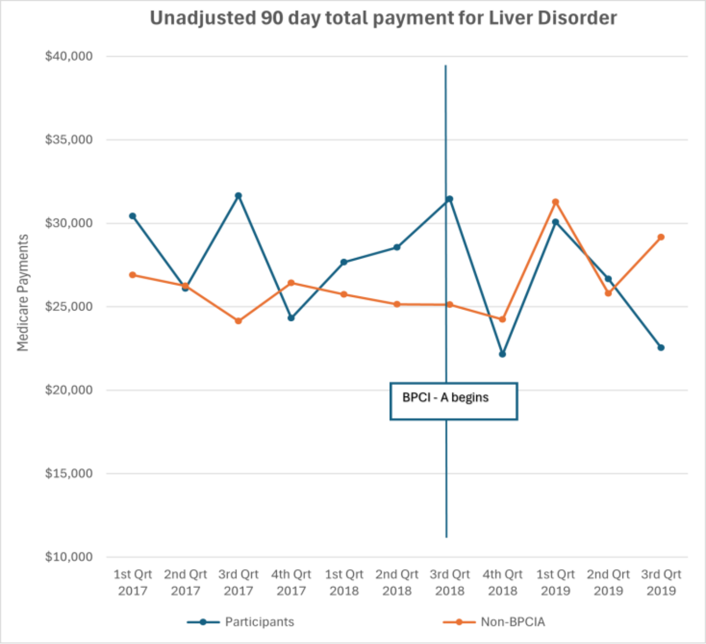

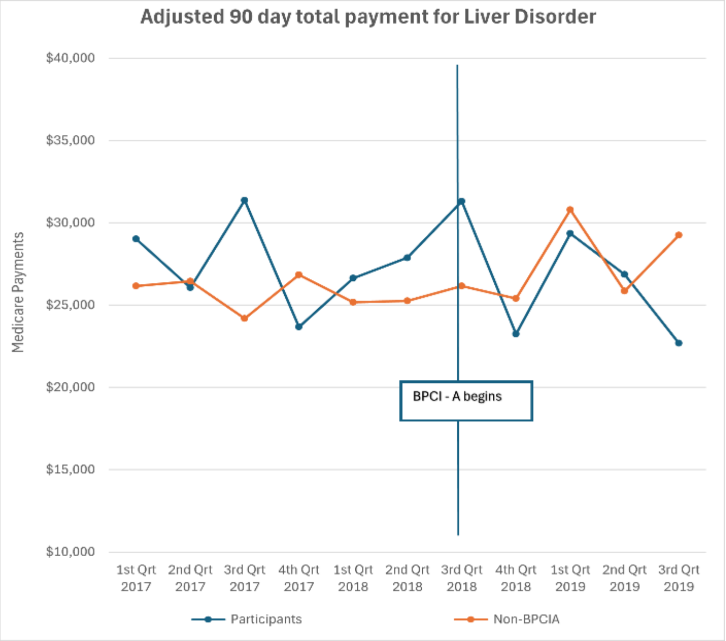

© 2024 Robbins KJ et al. *JAMA Network Open.*

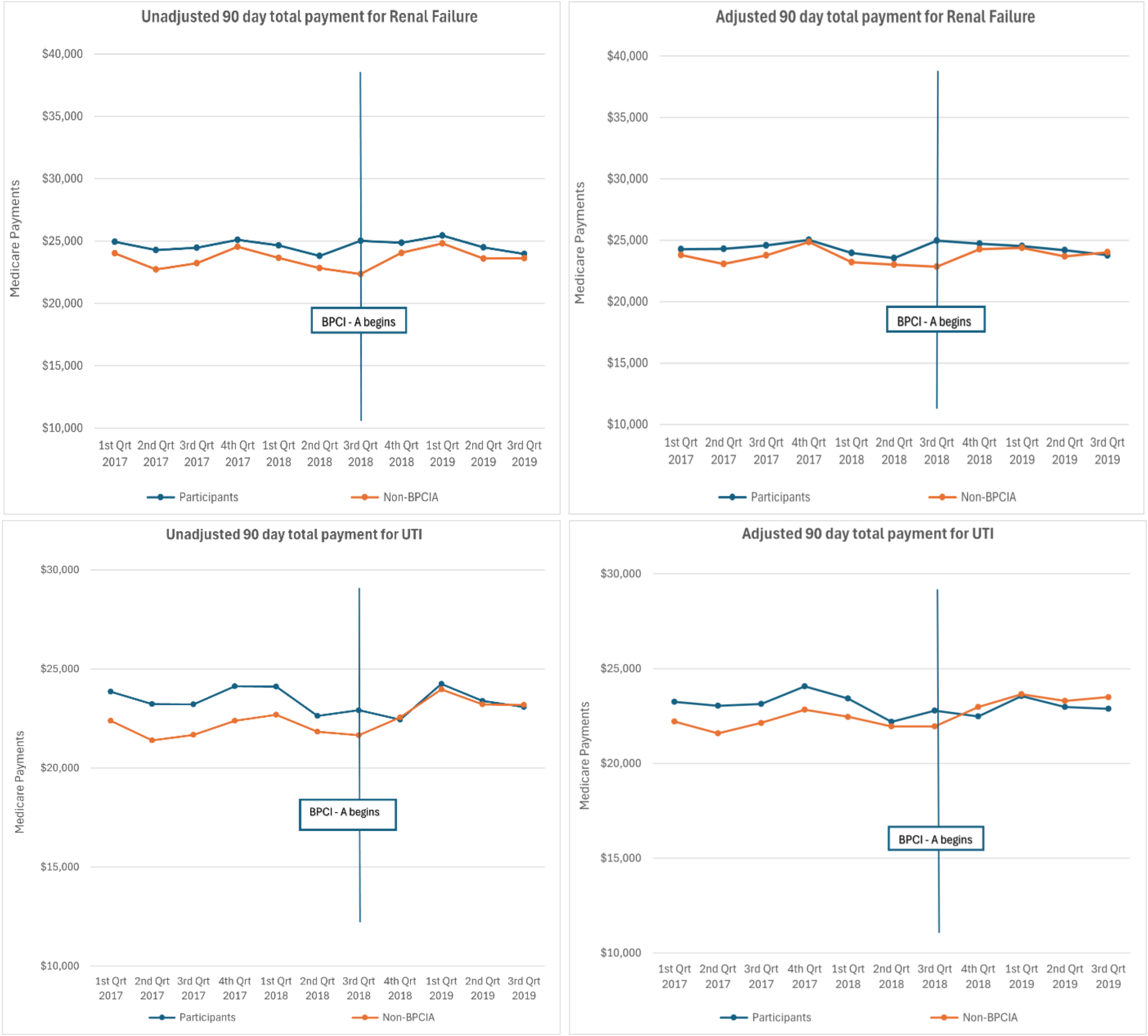

© 2024 Robbins KJ et al. *JAMA Network Open.*

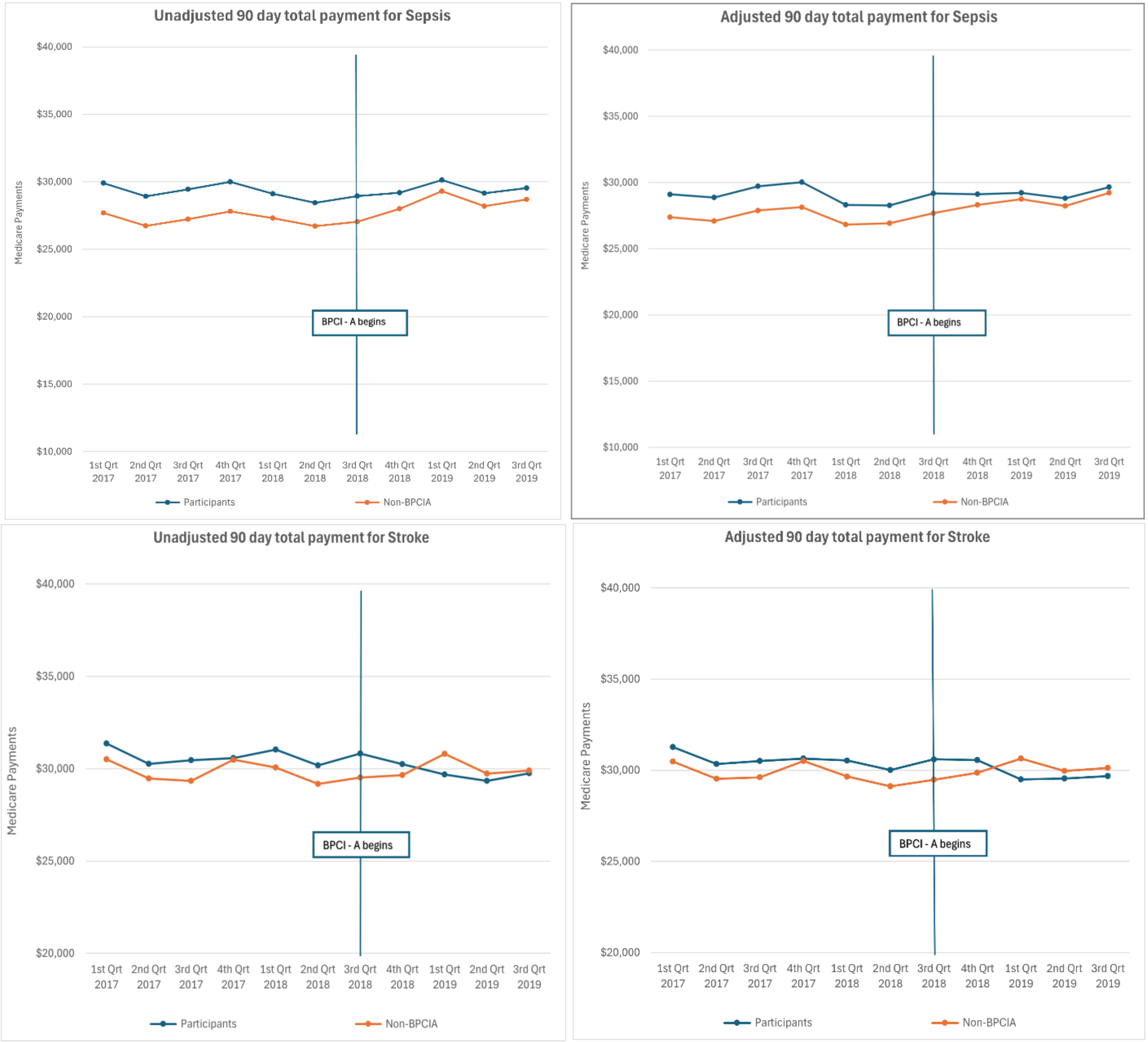

© 2024 Robbins KJ et al. *JAMA Network Open.*

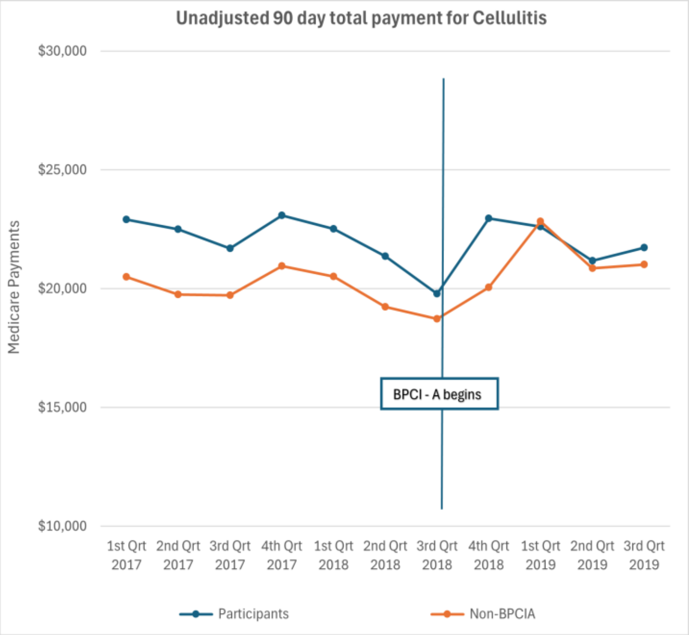

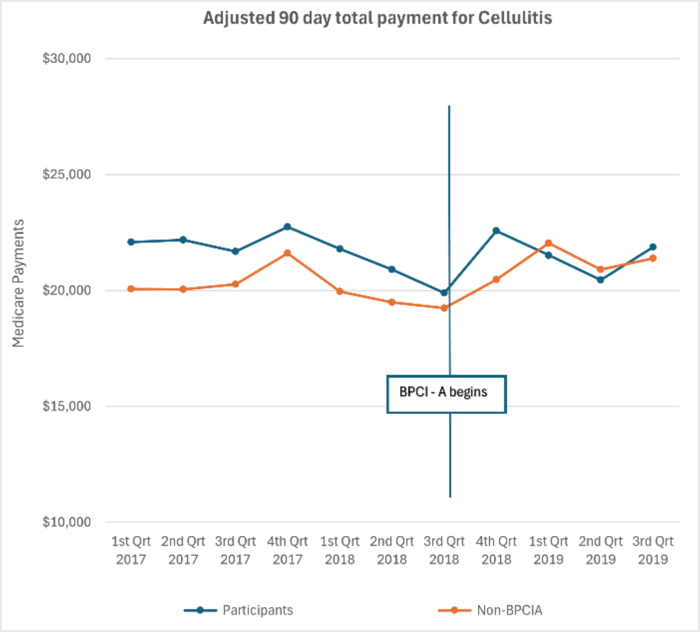

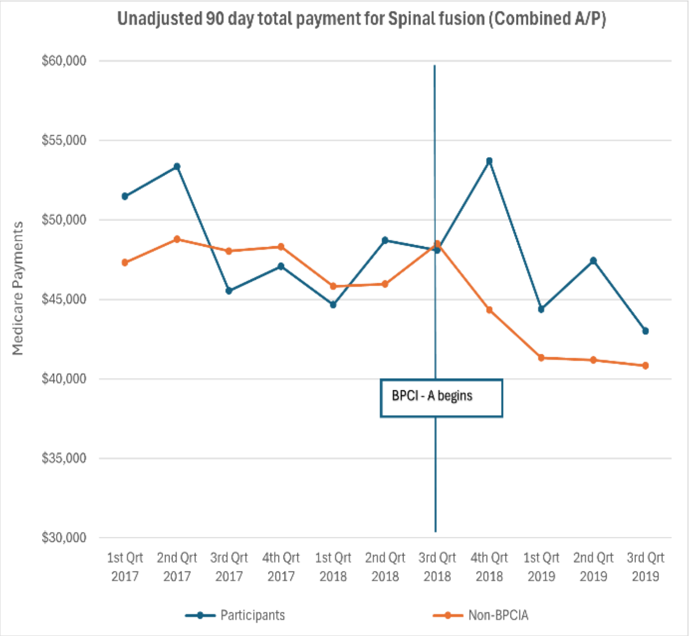

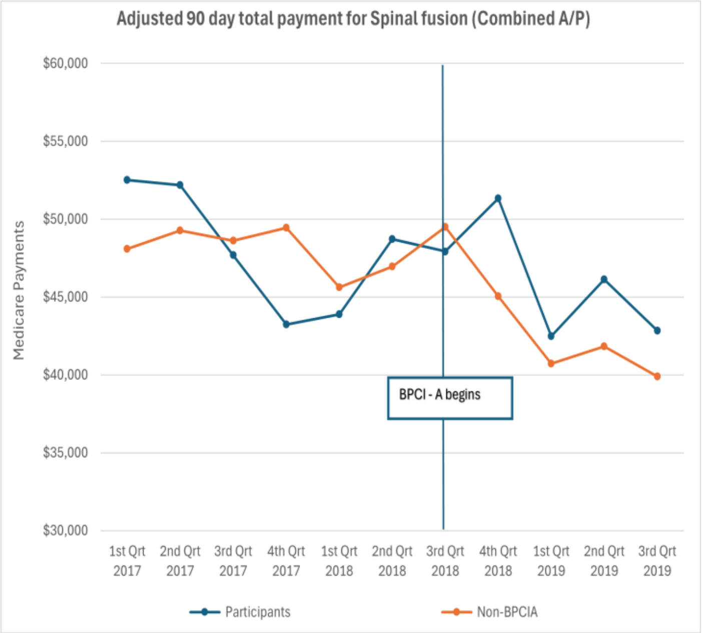

© 2024 Robbins KJ et al. *JAMA Network Open.*

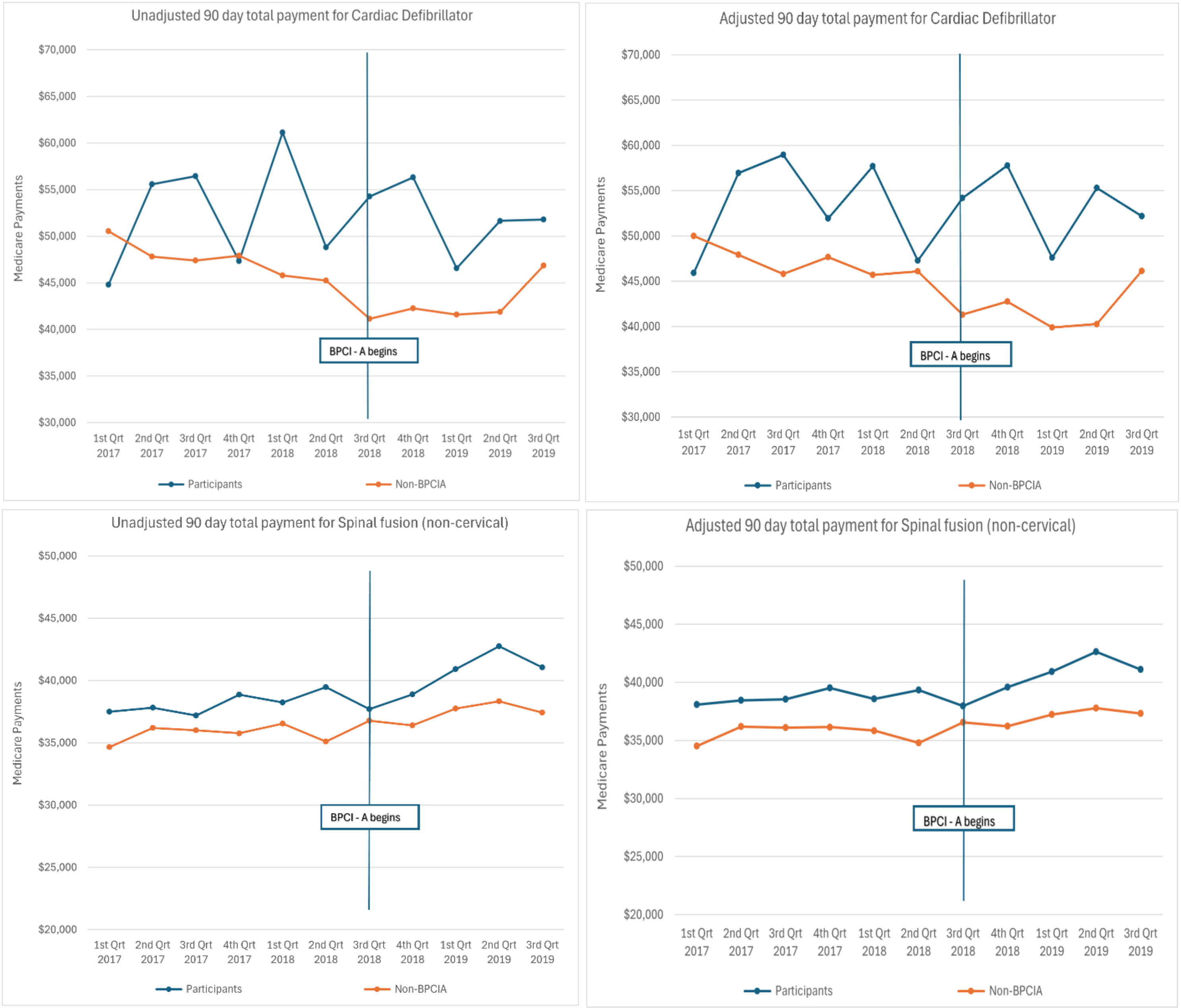

© 2024 Robbins KJ et al. *JAMA Network Open.*

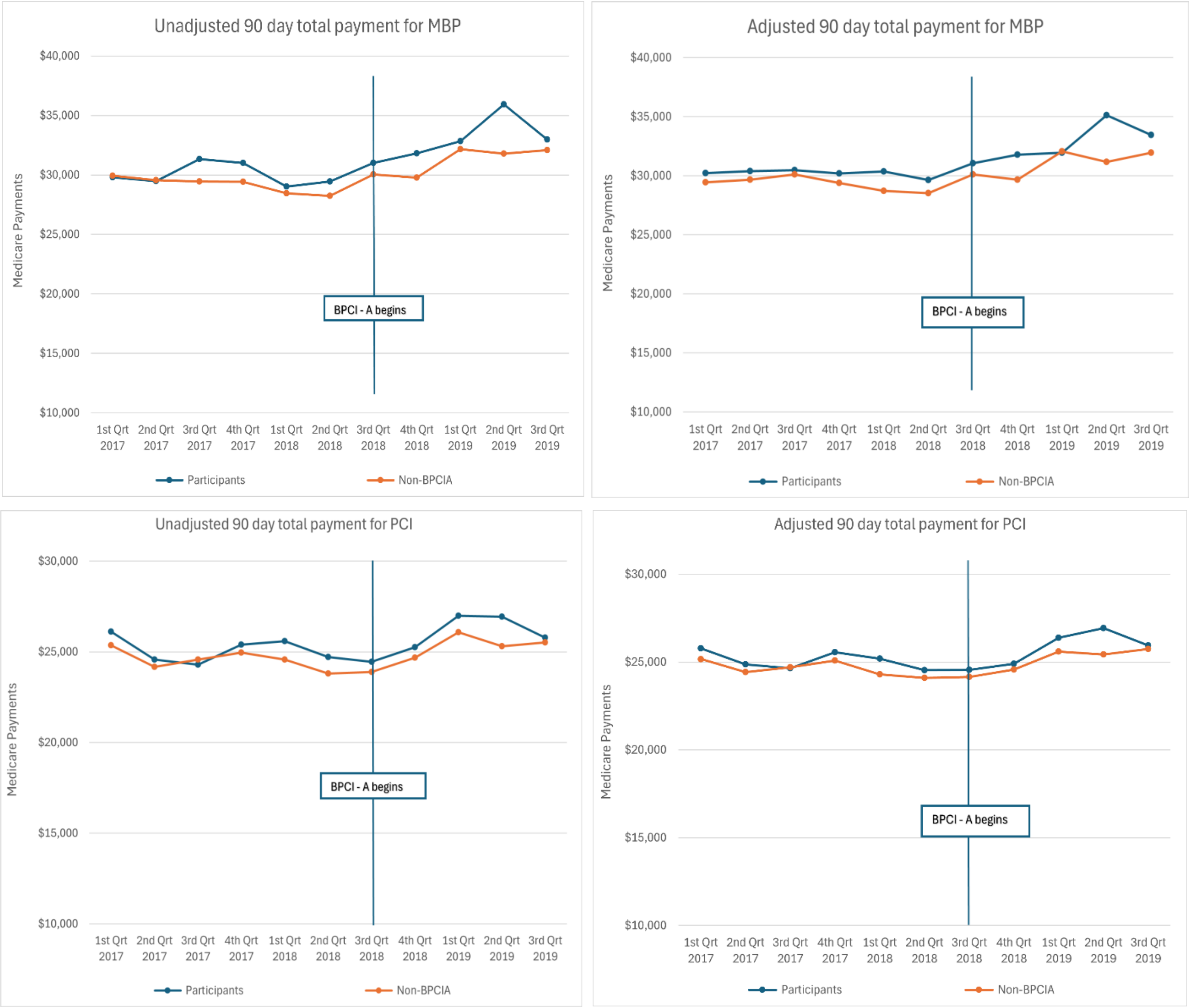

© 2024 Robbins KJ et al. *JAMA Network Open.*

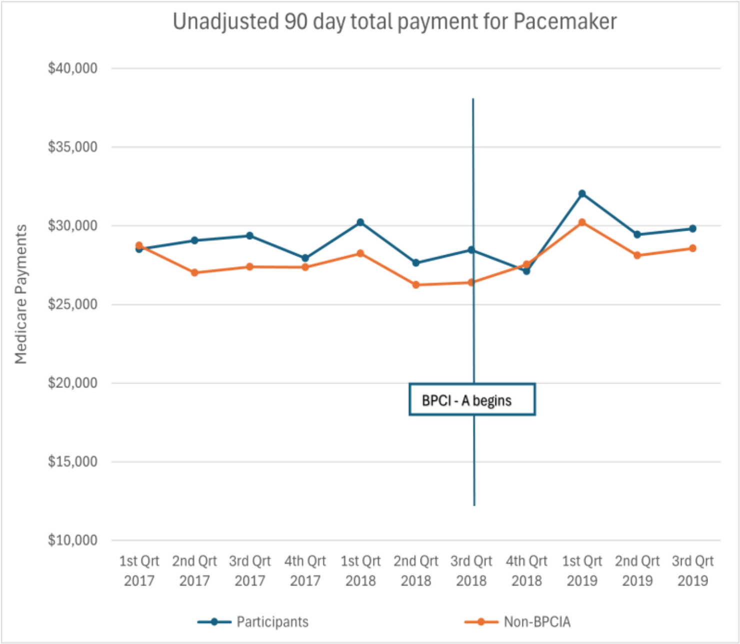

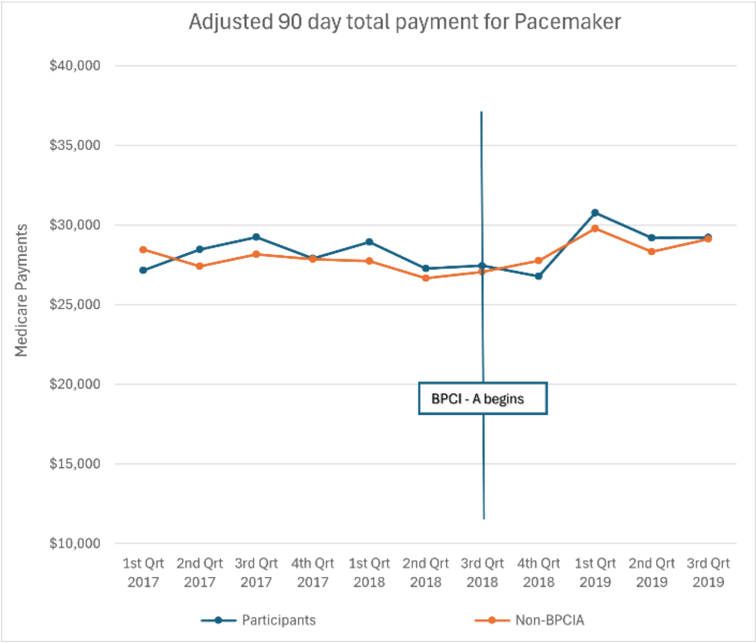

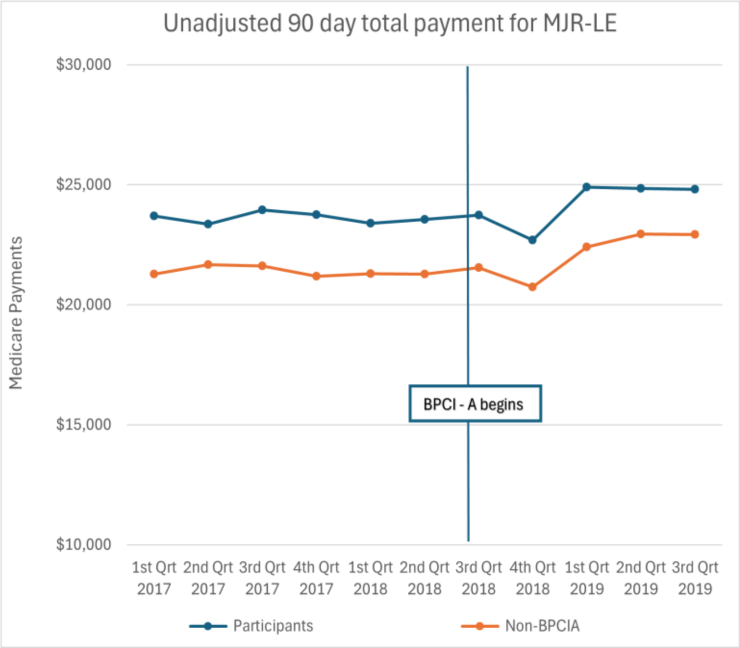

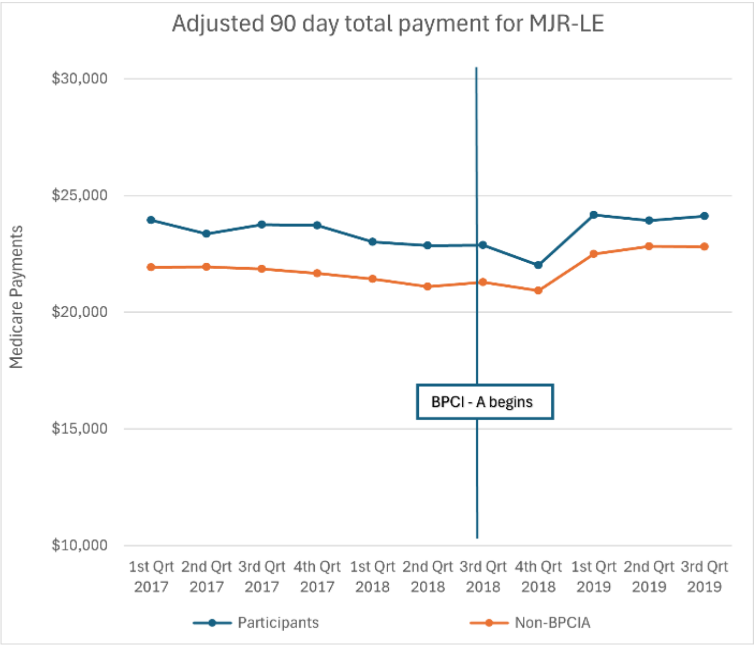

© 2024 Robbins KJ et al. *JAMA Network Open.*

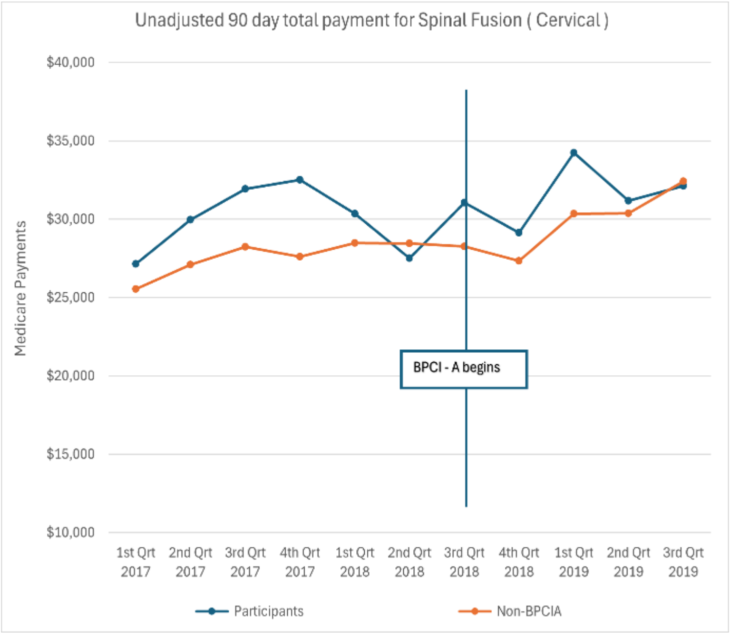

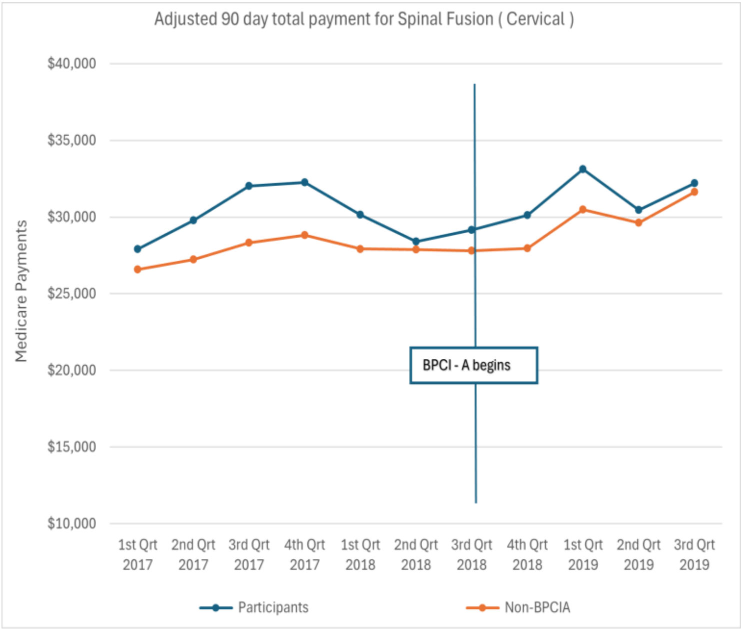

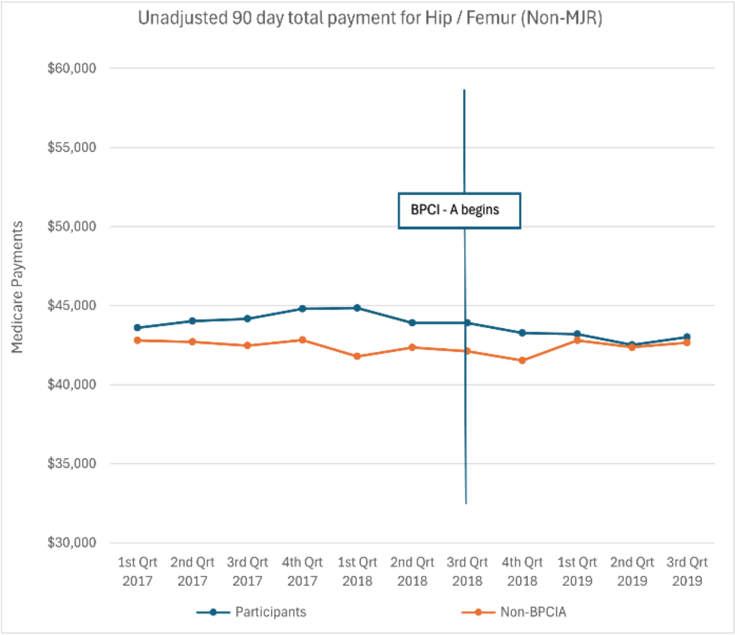

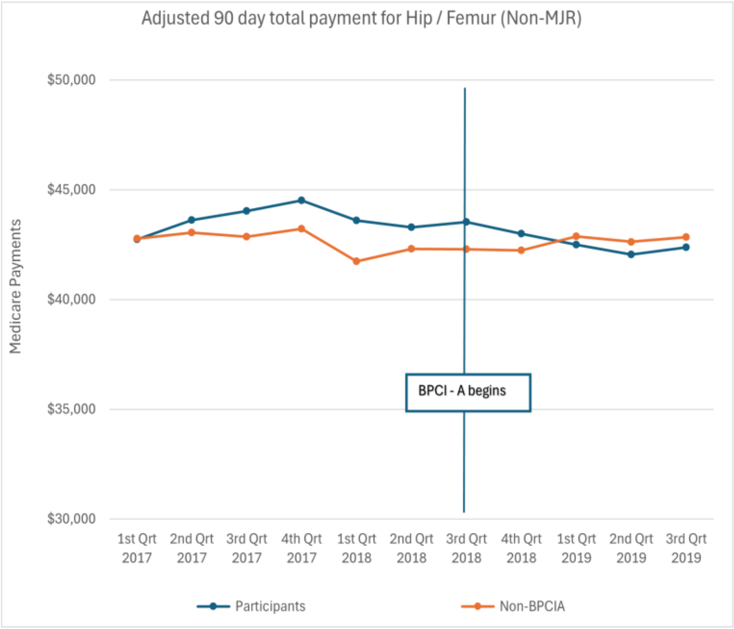

© 2024 Robbins KJ et al. *JAMA Network Open.*

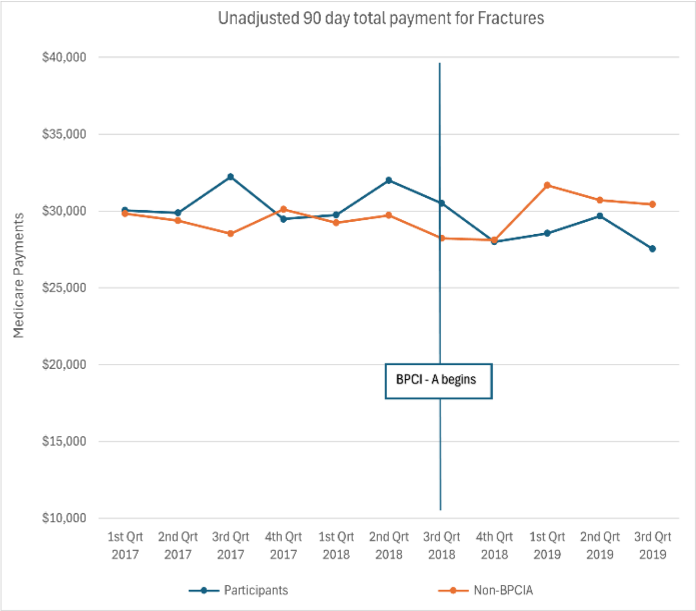

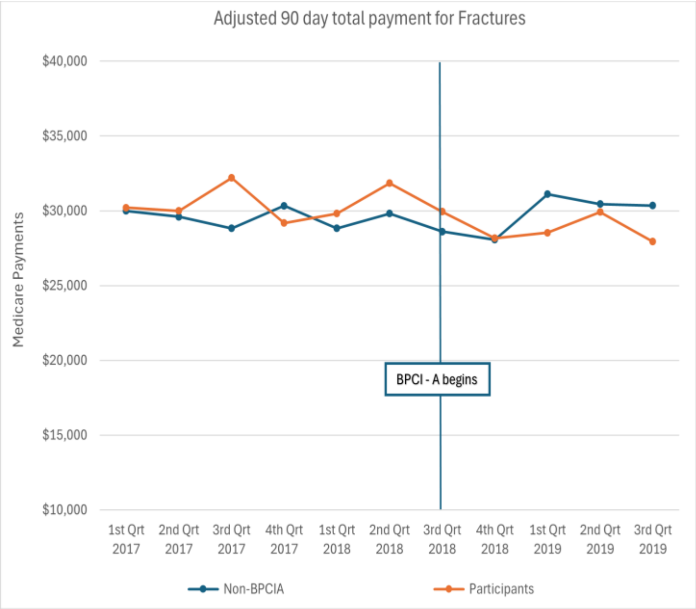

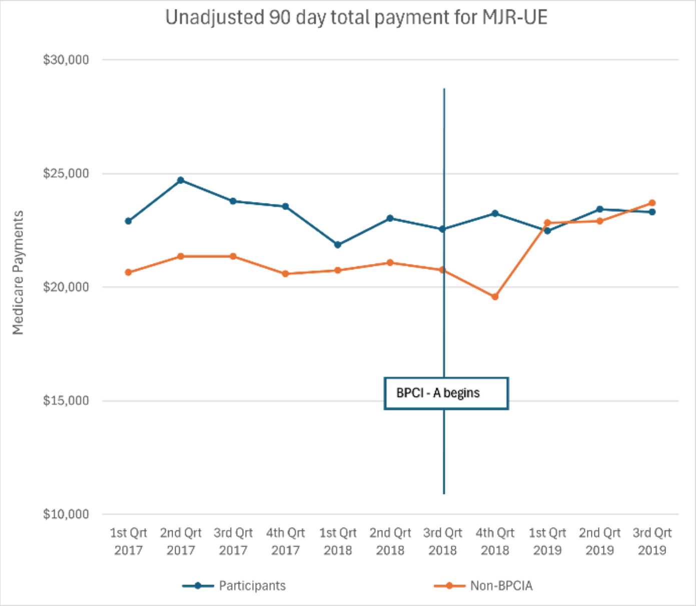

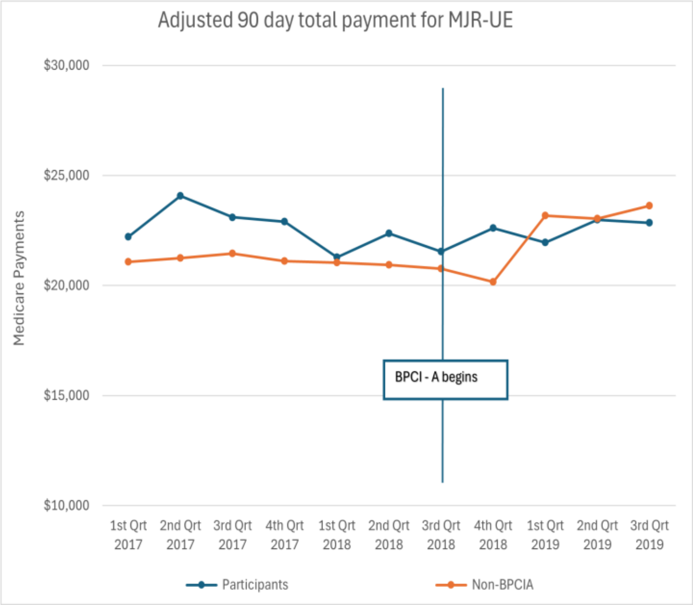

© 2024 Robbins KJ et al. *JAMA Network Open.*

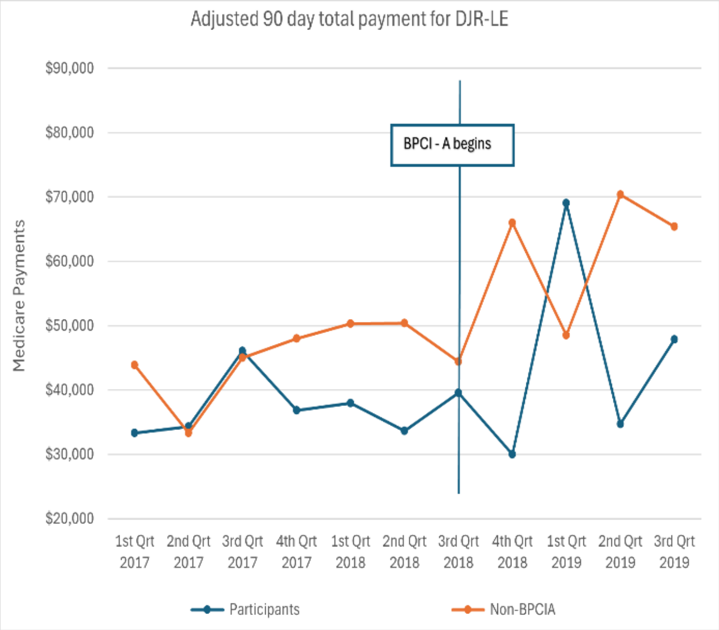

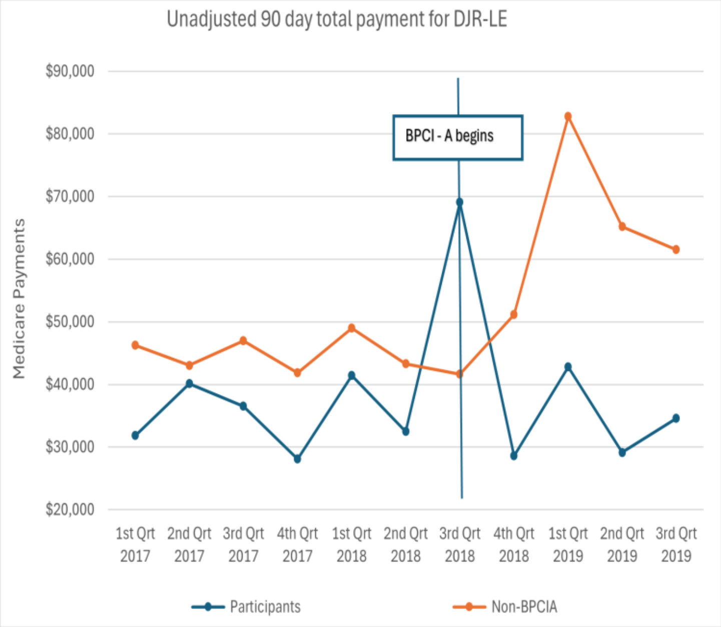

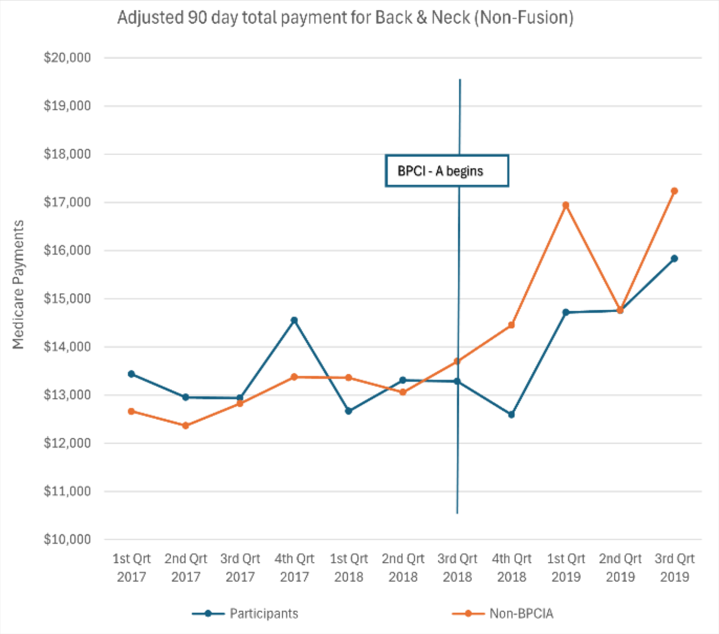

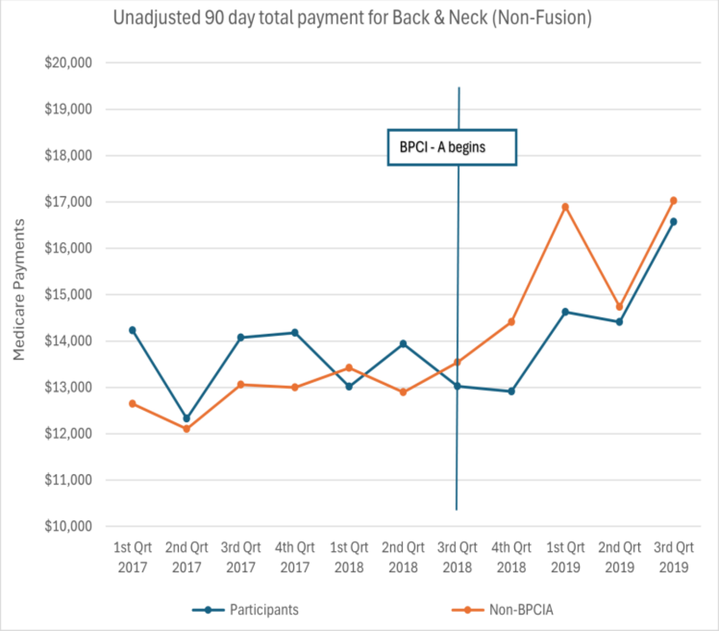

© 2024 Robbins KJ et al. *JAMA Network Open.*

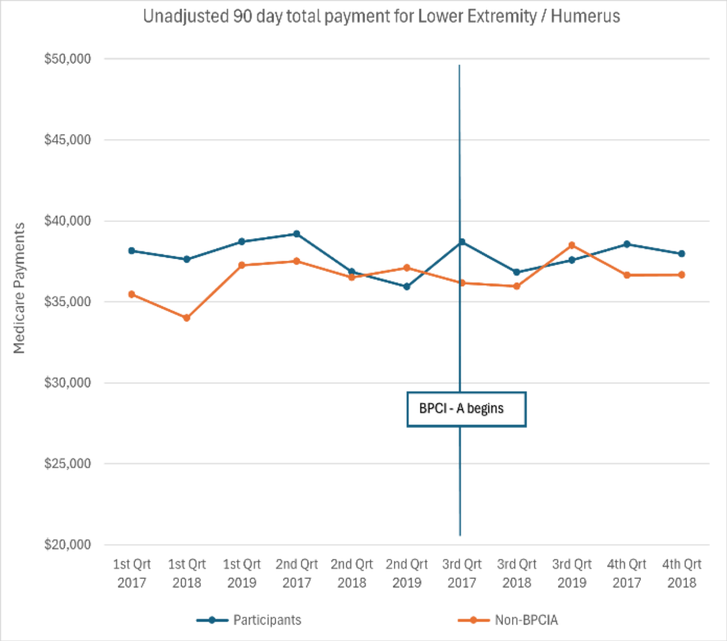

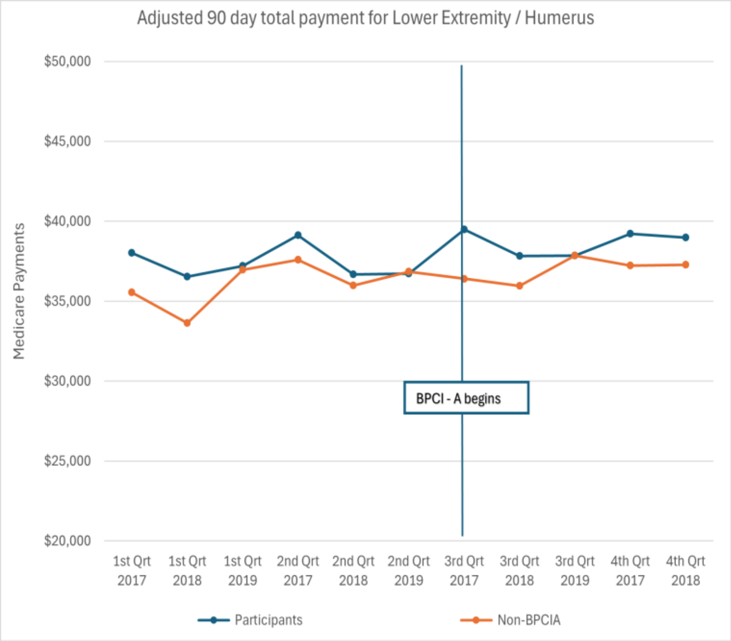

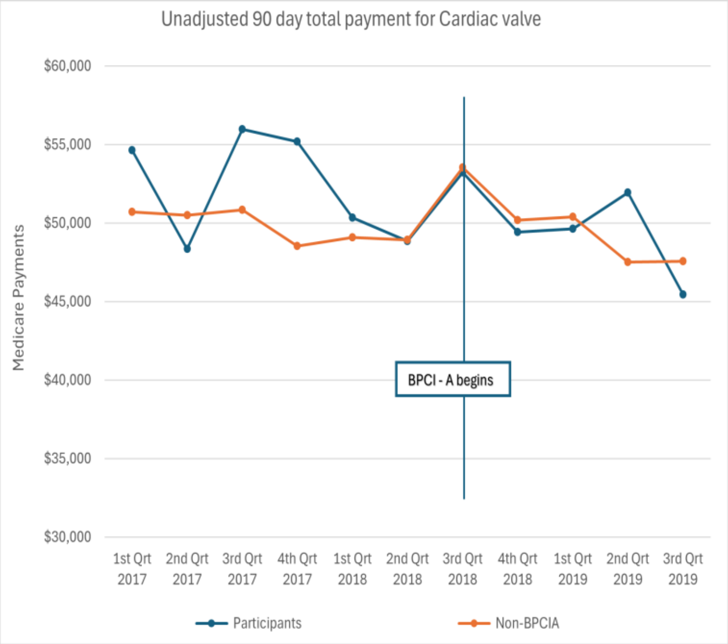

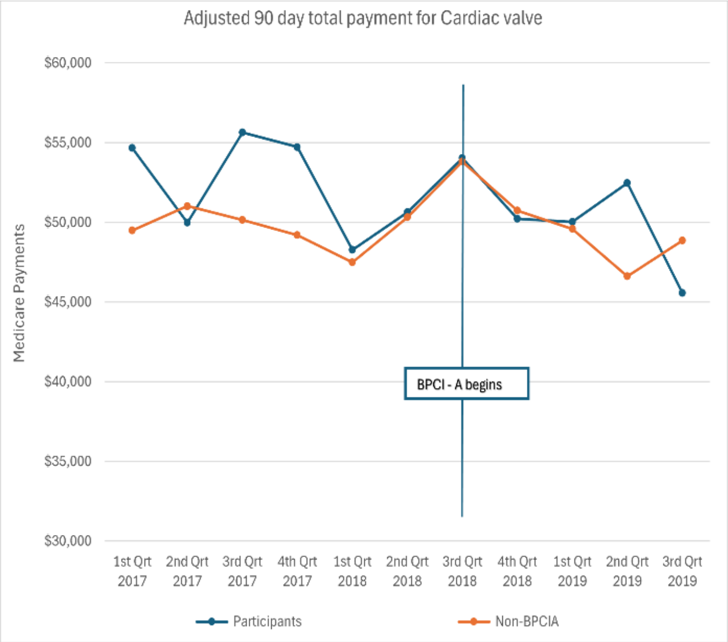

© 2024 Robbins KJ et al. *JAMA Network Open.*

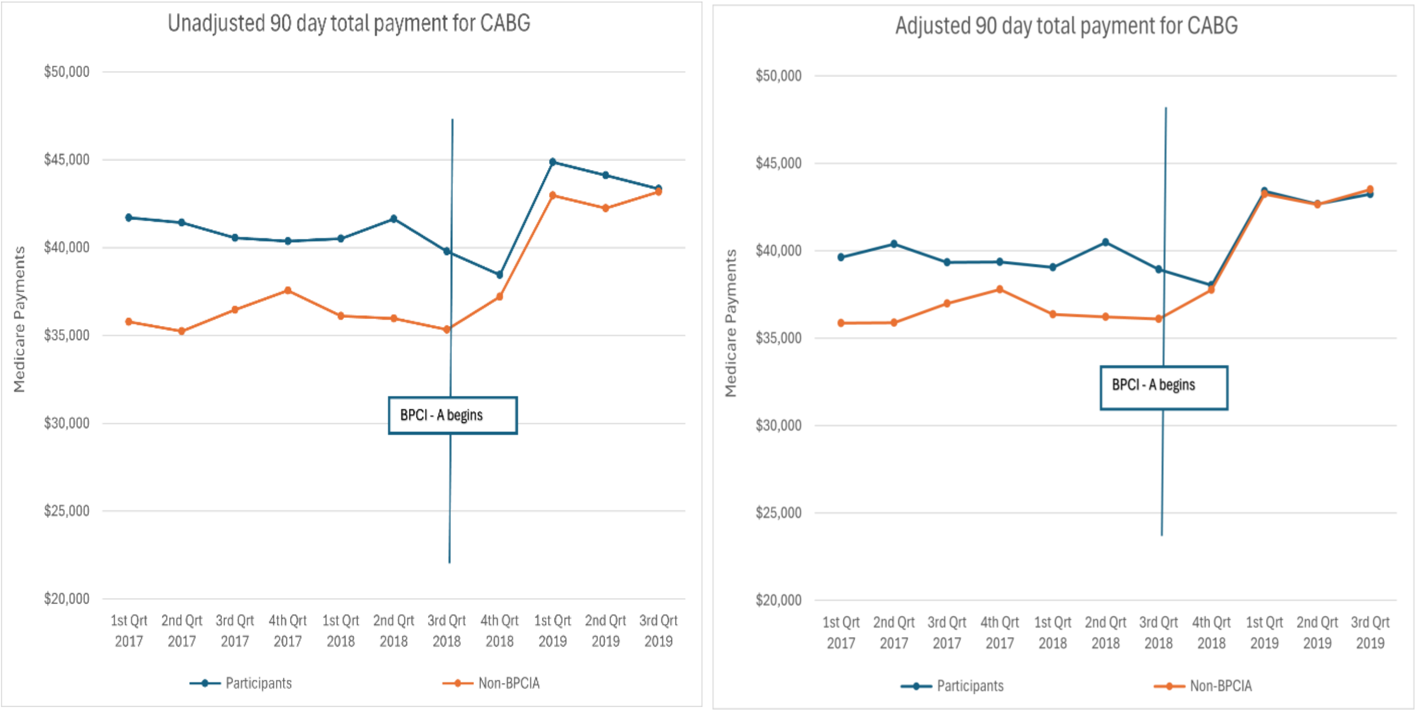

AMI = acute myocardial infarction. Arrhythmia = cardiac arrhythmia. Back / neck (non-fusion) = back and neck except spinal fusion. CABG = coronary artery bypass graft. CHF = congestive heart failure. COPD = chronic obstructive pulmonary disease, bronchitis, asthma. Defibrillator = cardiac defibrillator. DJR-LE = double joint replacement of the lower extremity. Fractures = fractures of the femur and hip or pelvis. GI hemorrhage = gastrointestinal hemorrhage. GI obstruction = gastrointestinal obstruction. HDAH = healthy days at home. Hip / femur (non-MJR) = hip and femur procedures except major joint. Liver disorders = disorders of liver except malignancy, cirrhosis, or alcoholic hepatitis.. Lower ext / humerus = lower extremity and humerus procedure except hip, foot, femur. MBP = major bowel procedure. MJR-LE = major joint replacement of the lower extremity. MJR-UE = major joint replacement of the upper extremity. PCI = percutaneous coronary intervention (inpatient). PNA = simple pneumonia and respiratory infections. SNF LOS = mean length of SNF stay for those discharged to SNF. SNF stay = percentage of patients with SNF stay after discharge. Spinal fusion (A/P) = combined anterior posterior spinal fusion. UTI = urinary tract infection.

© 2024 Robbins KJ et al. *JAMA Network Open.*
