# Supplementary material for: Medical and Surgical Episodes Among Hospital Participants in the Bundled Payments for Care Improvement–Advanced Program
Source: JAMA Netw Open. 2024 Dec 23;7(12):e2451792. doi: 10.1001/jamanetworkopen.2024.51792 (PMC11667361; doi:10.1001/jamanetworkopen.2024.51792)
Supplement: Supplement 2. — Data Sharing Statement [file jamanetwopen-e2451792-s002.pdf]

Data Sharing Statement

Robbins. Outcomes for Medical and Surgical Episodes Among Hospital Participants in the Bundled Payments for Care Improvement Advanced Program. *JAMA Netw Open*. Published December 19, 2024. doi:10.1001/jamanetworkopen.2024.51792

Data
Data available: No
